# Social Support of Patients with Type 2 Diabetes in Marginalized Contexts in Mexico and Its Relation to Compliance with Treatment: *A Sociocultural Approach*

**DOI:** 10.1371/journal.pone.0141766

**Published:** 2015-11-06

**Authors:** Clara Juárez-Ramírez, Florence L. Théodore, Aremis Villalobos, Aida Jiménez-Corona, Sergio Lerin, Gustavo Nigenda, Sarah Lewis

**Affiliations:** 1 Centre for Health Systems Research, National Institute of Public Health, Cuernavaca, Morelos, Mexico; 2 Centre for Nutrition Research, National Institute of Public Health, Cuernavaca, Morelos, Mexico; 3 Centre for Population Health Research, National Institute of Public Health, Cuernavaca, Morelos, Mexico; 4 Department of Ocular Epidemiology and Visual Health, Institute of Ophthalmology Conde de Valenciana IAP, México City, Mexico; 5 Directorate of Epidemiology, Ministry of Health, Mexico City, Mexico; 6 Department of Medical Anthropology, Centre for Research and Studies in Social Anthropology, Mexico City, Mexico; 7 School of Medicine, Morelos State Autonomous University, Cuernavaca, Morelos, Mexico; 8 Doctoral Student, Health Services & Policy Analysis, University of California, Berkeley, California, United States of America; Institute of Endocrinology and Metabolism, IRAN, ISLAMIC REPUBLIC OF IRAN

## Abstract

**Objective:**

This study aimed to describe the ways social support works in the daily life of patients with type 2 diabetes living in conditions of social and economic marginality, in order to understand how that support relates to treatment compliance.

**Methods:**

Sequential mixed methods research was used. The sample of patients was obtained from primary health care units and selected considering regional representativeness, and levels of morbidity and mortality for type 2 diabetes.

**Results:**

Results point to the nuclear family as the main source of support. Regardless of the area of residence, four main dimensions of support were identified: economic support, help with treatment compliance, emotional support, and material aid.

**Conclusions:**

We conclude that the support network assists the patient in different ways and helps cope with the disease, but in conditions of social and economic marginality, does not guarantee the quality of attention nor enable the self-management of treatment.

## Introduction

In Mexico, as around the world, the number of people with chronic non-communicable diseases such as type 2 diabetes is steadily increasing. There are about 347 million of people with diabetes; by 2030 this disease will be the seventh leading cause of death in the world. More than 80% of deaths from diabetes are registered in low- and middle-income countries [1–4]. The development of the disease is related to unhealthy lifestyles including poor eating habits, sedentary lifestyle, and genetic factors. Diabetes can lead to serious complications due to comorbidities (dyslipidemia, hypertension) and associated consequences (blindness, kidney disease, amputations) at advanced stages [5]. Diabetes has no cure but can be controlled by following prescribed treatment and lifestyle change; the goal is that the patient be able to self-manage the disease. However, only a small percentage of people comply with medical recommendations. In Mexico, recent data indicate that only 26.8% of persons with a diagnosis of diabetes modified their eating habits and 10% reported exercising as part of the treatment [6].

The World Health Organization has indicated that public policies addressing chronic diseases have failed [7]. One possible explanation is that the biomedical paradigm underlying the design of prevention and care programs does not recognize adequately social and cultural determinants; it considers the patient as an isolated entity and ignores how social relationships are interwoven and function around the sick person [8]. It also blames the patient for his or her lifestyle and does not address the impact of socio-economic status in the generation of vulnerable social environments, nor does it relate its influence to risk behaviors, despite evidence between being diabetic and belonging to the poorest and most excluded social strata [9].

Literature from the social sciences helps to understand the immediate environment of these patients. At the individual level, the greatest impact occurs via emotional experience. After being diagnosed, the patient must modify his or her everyday practices and adheres to treatment to make it effective [10]. According to Bury, a chronic disease represents a disruptive event that permanently affects everyday life. Patients face a critical situation that breaks apart their self-structure as constructed before the event. The sick person re-signifies the experiences of daily life and rearranges the spheres of common sense, personal history, and social and material resources [11]. With regard to social resources, there is evidence on how support, social networks and social capital impact patients. Those concepts emerge from different perspectives on the fabric of social relations and refer to resources perceived as natural, but in reality depend on the position that a person occupies in society. Social support is understood as an individual’s hypothetical perception of his or her available social resources [12]. This perception defines the potentiality of the “significant social networks” that people have to solve their difficulties within their micro-social environment. On the other hand, the network is the sum of all the relationships perceived as significant; it can be analyzed by its attributes of structure (density, connectivity, porosity) and performance (accessibility, social bond, type of exchange) [13,14]. Finally, social capital is an intangible good comprised of the set of informal relationships people have, as well as those of trust and cooperation [15].

Studies on social support point to the benefits patients obtain from knowing they are part of an established network. For Cassel, there is a psychological component of social relations related to being integrated into a support group. Having a sense of belonging to a group and knowing one can count on that group in extreme situations positively affects health [16]. Cobb showed that people who receive support develop a notion of belonging to a social network, which acts as a protective factor and reduces the stress entailed by adverse events in everyday life [17, 18].

Specific studies on populations with diabetes, hypertension, and cancer [19–21] have tested hypotheses about protective factors. These studies agree on the importance of social networks in providing support during the relevant events that occur to people with chronic disease. Other findings [22, 23] stress the importance that persons affected by these diseases not only perceive a support network close to them, but also be willing to provide support, as the feature that enables mutual aid is *reciprocity*.

Finding a way to achieve patients’ treatment compliance is a challenge for the health system. In addition to the negative effects the disease has on individual health, its medical care involves substantial economic investment on behalf of the State [24].

This paper presents partial results of an extensive study with multiple goals, including describing the ways in which social support works in the daily life of public health sector patients with type 2 diabetes, in the context of social and economic marginality in Mexico. Research Question: How do social resources in patients with type 2 diabetes support adherence to treatment?

## Methods

### Research team

Two medical anthropologists served as principal investigators and coordinated the entire research process of (CJR, SeL). In the data collection stage two teams of anthropologists and nurses were trained. The first team consisted of five women and one man: a physician, three anthropologists, one political scientist and the co-principal investigator. The second team comprised six women and two men (two nurses, three anthropologists and one political scientist). All team members received training to standardize their knowledge about project objectives, data collection techniques and field work procedures. Additionally, three anthropology students of indigenous origin who spoke the local language were hired as translators and data collectors in the indigenous study areas. Their training was identical to the other teams’ and included a special emphasis on the meaning of the questionnaire and interview guide questions so the information would be appropriately translated when collecting and entering the data. They were instructed in how to ask the questions taking into consideration local cultural modalities. This training was especially important due to illiteracy among rural and indigenous populations; the latter may not necessarily speak Spanish.

All research team members had previous data collection experience, both for surveys and qualitative interviews. None of the interviewers had a previous relationship with the interviewees. The team received training on ethical issues for working with indigenous populations. Principal Researchers were trained in Ethics with the online course: CITI, University of Miami. The primary care unit authorities had no influence on the selection of respondents, nor were they present during interviews.

### Study design

The methodological design used a sequential mixed methods approach. These types of methods employ a technique which guides each subsequent phase of inductive investigation [25]. Given the lack of information on the topic, the first step of this study quantitatively characterized the everyday experience of patients with diabetes. This inductive process resulted in preliminary findings which informed the qualitative portion of the study; it identified the themes that would be addressed in the interview guides, and helped defined the analytical sample [26].

Qualitative data were analyzed within the theoretical framework of Phenomenology. From a relational perspective, the qualitative information let us approach the experience of illness from the actor’s point of view [27, 28].

The sample of patients was purposive, with previously established inclusion criteria and comprised people with type 2 diabetes seen in public sector primary care units. These types of health care users often belong to the lowest social strata in the Mexican population. The inclusion criteria were as follows: a) be a user of public health services; b) have a diagnosis of type 2 diabetes of at least one year; c) be an active member of a mutual support groups (MSG); d) be at least 18 years of age; and e) have other members of the family with type 2 diabetes. Diversity was sought in terms of geography (North, Central, and Southern Mexico), type of population (including indigenous, regarded as a person who spoke a language other than Spanish), area of residence (urban, rural, and indigenous localities), and levels of morbidity and mortality from type 2 diabetes. Based on these criteria, the States of Coahuila, Chiapas, Guanajuato, Quintana Roo and Yucatan were chosen. All public primary medical care units in each of the selected States were identified and the relevant statistics of cases of type 2 diabetes at these facilities were reviewed. Next, a purposive sample of medical units with MSG for patients with diabetes running for at least one year was obtained. The resulting sample comprised twelve medical units in twelve distinct locations.

### Data collection

Data were collected in two stages between 2008 and 2012. In the first stage, an 89-item questionnaire was designed that explored nine themes: socio-demographic characteristics (employment, income and housing characteristics), knowledge of the disease, self-care, use of traditional medicine, the doctor-patient relationship, compliance with treatment, consumption of alcohol and tobacco, participation in the mutual aid support group, and family composition. A pilot test was conducted to validate the questionnaire and adjustments were made to suit all three location scenarios. The questionnaires were administered during a weeklong visit to each location. During the visit to the medical unit, data collection team members presented their credentials upon meeting patients interested in participating. Participants were presented with a letter of informed consent explaining the research objectives and what their participation would consist of. With illiterate respondents the interviewers filled in the questionnaires directly. In this stage 553 questionnaires were collected: 308 from urban areas, 126 from rural areas, and 119 from indigenous localities.

In the second stage, a subsample of people with type 2 diabetes was selected to incorporate a qualitative approach. An interview guide explored nine themes: disease onset; knowledge of diabetes; actions taken to treat the disease; use of traditional medicine resources; utilization of institutionalized medicine resources; the patient’s family environment; participation in the MSG; diseases/conditions and health care; and family, social support and MSG. We also piloted and adjusted the interview guide.

The process for the second stage was as follows: First, we briefly interviewed people selected through the questionnaire. After a preliminary analysis of these data we realized the necessity of delving more deeply into the research for indigenous respondents, who were under-represented in stage 1. As a result we decided to expand the sample to states which has highest indigenous population in the country. The subsample for the qualitative analysis consisted of 214 cases (65 from urban areas, 46 from rural areas and 103 from indigenous localities). Subsequently, for the in-depth interviews we selected a subsample we called *paradigmatic cases*, from this we selected 25 cases (5 urban and 20 rural/indigenous), based primarily on personal history, health care trajectory, experience as a sick person and ability to narrate the experience. Most of these cases were patients of indigenous origin who had experienced complications from the disease (amputation, impaired vision, kidney damage, etc.). We also videotaped biographical documentaries with these subjects; a public version of the videos (in Mayan, with Spanish translation) is available at the following link: http://lenguas.ciesas.edu.mx/corpora/Lenguas_mayas/maya/Media/Tihosuco2.mp4

Interviews were conducted in private spaces inside the primary health care units, and in patients’ homes when requested. Qualitative interviews with indigenous participants were conducted in their native language (either Maya or Tzotzil). As an extra measure of quality control, some of the interviews conducted in Maya and Tzotzil were chosen at random and translated by other locals. The interviews lasted 30 to 150 minutes and all were audio-taped. The saturation point of each explored category was discussed with the entire research team.

This stage was complemented by participant observation using a guide developed to document selected aspects related to medical unit infrastructure as well as the relationship between health personnel and patients. To better understand the context of the interviewees ethnographic reports were generated from notes taken during observation. CJR and SeL supervised all data collection activities.

### Data analysis

This paper only reports on the results related to social support.

Stage 1: With the quantitative results we developed a sociodemographic profile of participants to describe the circumstances under which social support was likely to occur. Of the 553 cases, 547 answered the questions related to social support. Data collected in the questionnaires were entered into a quantitative database using SPSS software. The variable of interest *is supported* (Yes, No) was constructed based on the question *in relation to your disease*, *do you have the support of*…? The “yes” category included relatives, friends and neighbors. Additionally, the variable *person who provides support* was assigned to one of three categories: “relative” (including parents, siblings, spouse, domestic partner, and children), “other relative” (including grandchildren and nephews), and “non-relative” (including friends and neighbors). The variable *type of support* was constructed from the question *how are you supported*? The type of support was subdivided into “economic, care, emotional (affective), material (including clothing and food)” and “other.” A bivariate analysis between the categorical variables of interest (*is supported*) and socio-demographic variables and characteristics of the disease (time of diagnosis and its consequences) was carried out. To explore the association between variables, Pearson’s chi square test of independence was used, as well as the likelihood ratio test, where appropriate. Later a Student t-test was estimated for mean age to identify significant differences between the two groups. (S1 and S2 Tables: Dictionary Quantitative Variables and Quantitative Data Base, Social Support).

Stage 2: The qualitative interviews were audio recorded and transcribed literally into a word processor. We then selected interviews from *paradigmatic cases* to manually develop the main categories and codes. Subsequently we used the software Atlas ti version 6 to separate out the discussion segments from the rest of the interviews, using the previously identified manually developed codes. Four different people coded the interviews and the tree containing all the codes is available in S1 Fig: Code Tree all Categories. However, this article only presents the results of a single category—social support—and four codes: type of support, perception of support, disability and need for support. The results were interpreted and organized into the following themes: a) *Economic support*: related to cash assistance; b) *Assistance with care and treatment*: linked to medical treatment within the domestic sphere; c) *Emotional support*: related to the feelings of the patient; and d) *Material aid*: all forms of aid provided in-kind as well as notions of reciprocity. [29–31]. (S2 Fig: Code Tree category IX).

Data collected through participant observation were used to write ethnographic reports, which served to understand the context in which health services were used, the quality of health education received in the MSG, and the relationship between health center users and medical staff.

Study findings were presented to participants in community assemblies involving documentary screenings and distribution of the videos to those who had participated. A copy of the video was also left in each participating primary care unit and another given to the health authorities.

### Ethics statement

The Research Protocol, the instruments and the consent procedure, was approved by the committees of Ethics and Research of the National Institute of Public Health of Mexico on March 11, 2009. All participants gave written or verbal informed consent to participate in the research (verbal was audio recorded). Informed consent was translated in their original language (indigenous case). The Declaration of Helsinki-Ethical Principles for Medical Research Involving Human Subjects, were considered for this study (www.wma.net).

## Results

### The institutional context of public health care

Based on our observations we got to know the profile of public health services users and their difficulties in getting medical care. Here we describe the most relevant barriers, related to social support and adherence to treatment.

Most were women belonging to the most disadvantaged social strata, whose only chance of care came from being beneficiaries of the System of Social Protection in Health. This health insurance covers care of type 2 diabetes, but not every type of treatment that may be required by complications of the disease.

#### Organizational aspects of medical units

There were frequent flaws in the provision of drugs; after consultation, patients were not given prescription medications. Also, none of the observed units had a laboratory for routine patient examination. In such cases, patients are forced to purchase resources with their own money to avoid interruption of treatment. These patient expenditures are not only spent on drugs but also on periodic laboratory tests. For people living on a subsistence economy, like the indigenous population, an additional expense can be an economic pressure that significantly affects household spending. There was a better chance of receiving care in urban settings, where clinic hours extended to weekends and health workers were continuously available. Away from urban centers, however, the operational quality of the medical units decreased. In particular, indigenous localities had more drawbacks, both in opening hours and availability of health workers.

In all visited units, patients were receiving educational sessions for health care; the goal was to teach them how to manage treatment for type 2 diabetes and control the disease. In the educational sessions, called “mutual support groups”, patients were accompanied by family members, usually the wife for male patients and daughters or friends in the case of female patients. During these sessions the treatment recommended by the health care team was reinforced and focused on the following three aspects: a) eliminate excess carbohydrates, fats and sugars; b) take medications at the right time; and c) perform physical activity.

Regardless of the types of residence and patients, these recommendations were the same for everyone. Frequently, patients were unaware of certain types of food recommended during educational sessions. The topic of exercise was not motivating nor understood as an important part of treatment, not because they disliked the idea of exercise itself, but because how sports and exercises was demonstrated taking place in the gym (as recommended in the information sessions) was not part of the everyday way of life of this social group. This was more common among the indigenous population and in rural areas than in urban areas. There were no local adaptations as to the kind of foods and exercise recommended. On the other hand, the physical activity of patients throughout their daily life was not recognized as “exercise”, despite the physical work that is common in rural and indigenous settings. For example, men will grow corn and carry firewood, while women bring water to their homes from long distances and wash clothes by hand.

### Results of quantitative analysis

#### Characteristics of Participants

Of 553 persons surveyed, 82.6% were women and 17.4% were men and the average age was 56 years old. Of all respondents, 24.3% did not know how to read and write, while 68.3% had a basic level of education. Given that the majority of respondents were women and had minimal schooling, most (80.1%) declared performing housekeeping duties and only 8.2% said they lived alone. Table 1 shows differences in sociodemographic and behavioral variables according to type of locality. The proportion of women in indigenous (86.1%) and rural (88.7%) areas was higher than in urban localities (73.7%, p<0.0001). Illiteracy was also higher in indigenous (39%) and rural (25%) localities than in urban areas (8.5%, p<0.0001). Significant differences between the three groups were also found for behavioral variables. Individuals from rural localities rated self-care more highly (p<0.0001), and people from urban areas had a higher family history of diabetes (p<0.0001). (Table 1).

**Table 1:** Sociodemographic characteristics and behavior of interviewed patients according to localities.

	Indigenous localities	Rural area	Urban area	P value^a^	Total
	n (%)	n(%)	n(%)		n(%)
	195 (35.26)	168(30.38)	198(34.36)		553(100)
**Socio-demographic characteristics**					
**Age (years), mean (s.d.)**	56.5 (11.0)	56.6 (10.7)	56.7 (10.7)		56.6 (10.8)
**Sex (women)**	168 (86.1)	149(88.7)	140(73.7)	<0.0001	457(82.6)
**Schooling**					
Illiteracy	76(39)	42(25)	16(8.5)	<0.0001	134(24.3)
Elementary	107 (54.9)	125(74.4)	145(76.7)		377(68.3)
Higher than elementary	12(6.1)	1(0.6)	28(14.8)		41(7.4)
**Occupation**					
Housekeeper	149(86.1)	133(80.6)	129(73.1)	<0.001	411(80.1)
Farmer or trader	22(11.6)	4(2.4)	19(10.2)		45(8.3)
Worker	7(4.0)	28(17.0)	2(1.1)		37(7.2)
Professional or other	12(6.3)	2(1.2)	37(20.0)		51(9.4)
**Knowledge of the disease** ^b^	2(1.0)	5(3.0)	4(2.3)	0.393	11(2.0)
**Self-care** ^c^	17(8.7)	25(14.8)	1(0.5)	<0.0001	43(7.8)
**Use of traditional medicine** ^d^	5(2.6)	3(1.8)	5(2.6)	0.837	13(2.4)
**Satisfied with their medical attention**					
Yes	170(90.9)	152(91.6)	172(91.0)	0.124	494(91.1)
Sometimes	13(6.9)	14(8.4)	12(6.4)		39(7.2)
**Consumption of alcohol**	18(9.2)	14(8.3)	14(7.4)	0.803	46(8.3)
**Consumption of tobacco**	6(3.1)	8(4.8)	15(7.9)	0.1	29(5.2)
**Family composition (lives alone)**	14(7.2)	14(8.4)	17(9)	0.821	45(8.2)
**Family history of diabetes**	101(51.8)	130(77.4)	157(82.6)	<0.0001	388(70.2)

^a^ Pearson’s chi square test or likelihood ratio test.

^b^ Knowledge of the disease considered if it meets three characteristics: known that diabetes has no cure, knows the three basic measures o control diabetes (diet, exercise and medication) and if thye know three diabetes complications (visual impairment, kidney failure and foot ulcers).

^c^ It includes cases where adequately comply with medication, with a special diet, exercise and control strips.

^d^ It is including that on occasion have attended the healer to treat diabetes and in herbal treatment included.

Of the total number of patients surveyed, 547 (98.9%) answered the questions on social support. Most of those who responded yes to receiving support were women (82.6%). Overall, 447 (81.7%) declared having received help in caring for their disease and in almost all cases the patient’s relatives (93.5%) provided it (Table 2). A significant relationship was observed between this outcome and marital status (73% in the group receiving support and 57.6% in the unsupported group lived as a couple, p = 0.002). Additionally, of the 418 individuals living with family, 93.5% reported receiving support, whereas their counterparts did not (83.3%, p = 0.002).

**Table 2:** Socioeconomic characteristics of surveyed patients according to “receives support or not to care for the disease”.

		Did receive support		p value^a^
		Yes	No	
		n(%)	n(%)	
	Total	447(81.7)	100(18.3)	
**Sex**	Men	78(17.5)	17(17)	
	Women	369(82.6)	83(83)	0.915
**Age, mean (S. D.)**		56.6(10.9)	56.4(10.6)	0.8903
**Age group**	21–49 years	110(26.1)	24(26.1)	
	50–64 years	205(48.7)	44(47.8)	
	65–86 years	106(25.2)	24(26.1)	0.982
**Marital status**	Single	36(8.1)	6(6.1)	
	Separated/Divorced	26(5.8)	13(13.1)	
	Widowed	58(13)	23(23.2)	
	Married/Cohabitation	325(73)	57(57.6)	0.002
**Literacy**	Iliteracy	108(24.3)	25(25)	
	Elementary	311(69.6)	61(61.0)	
	Higher than elementary	27(6.0)	14(14.0)	0.067
**Occupation**	Housekeeper	340(77.1)	67(67.7)	
	Farmer or trader	32(7.3)	13(13.1)	
	Worker	32(7.3)	5(5.1)	
	Professional or other	37(8.4)	14(14.1)	0.052
**Beneficiary from a government assistance program**	Yes	197(45.0)	48(49.0)	
	No	241(55.0)	50(51.0)	0.472
**People the patient lives with**	Lives alone	29(6.5)	16(16.2)	
	Lives with a relative	418(93.5)	83(83.8)	0.002

^a^ Pearson’s chi square test, likelihood ratio test or Student t-test

A total of 26.9% of persons reported having lived more than 12 years with type 2 diabetes and a large proportion already had consequences associated with the disease (32.4%). The main problem was loss of vision (83.6%), followed by neuropathy (10.7%) and diabetic coma (6.8%). Eight cases (4.5%) had experienced amputation of the lower limbs; these cases were persons of indigenous origin who did not speak Spanish (the language in which health services are provided). Their stories reflect the influence of ethnic condition in the delay in seeking medical care and poor adherence to treatment; in addition, their physical disability impeded them from attending medical consultations. No significant differences were found between those receiving and not receiving support for the outcomes described above (Table 3).

**Table 3:** Time elapsed after diagnosis of diabetes and complications of the disease.

	Received support			
	Yes	No	Total	
	n(%)	n(%)	n(%)	p-value^a^
Total	447(81.7)	100(18.3)		
3 years	128(29.2)	22(22.9)	150(28.0)	
4–12 years	192(43.7)	49(51)	241(45.1)	
>12 years	119(27.1)	25(26)	144(26.9)	0.357
**Did have complications**				
No	300(67.3)	69(69)	369(67.6)	
Yes	146(32.7)	31(31)	177(32.4)	0.738
**Type of complication**				
Ulcers	17(11.6)	2(6.5)	19(10.7)	-
Amputations	7(4.8)	1(3.2)	8(4.5)	
Nephropathy	5(3.4)	2(6.5)	7(4.0)	
Visual impairment	121(82.9)	27(87.1)	148(83.6)	
Diabetic coma	9(6.2)	3(9.7)	12(6.8)	

^a^ Pearson’s chi square test or likelihood ratio test

Regardless of the area of residence (urban, rural, or indigenous locality), respondents agreed on four main dimensions of assistance to cope with their disease: i) economic support (51.5%); ii) assistance in complying with treatment (25.1%); iii) emotional support (20.8%); and iv) material aid (11%). (Table 4). (S2 Table: Quantitative Data Base, Social Support).

**Table 4:** Person who provided the assistance and type of support received.

	Indigenous localities	Rural area	Urban area	Total
	n(%)	n(%)	n(%)	n(%)
Total	147(32.9)	150(33.6)	150(33.6)	447
**Person who provided the assistance**				
Relative^a^	135(91.8)	141(94)	142(94.7)	418(93.5)
Other relative^b^	7(4.8)	8(5.3)	2(1.3)	17(3.8)
Non-relative^c^	3(2.0)	9(6.0)	1(0.7)	13(2.9)
**Type of support**				
Economic	82(55.8)	75(50)	73(48.7)	230(51.5)
Care for treatment	34(23.1)	38(25.3)	40(26.7)	112(25.1)
Emotional	23(15.6)	36(24)	34(22.7)	93(20.8)
Material	19(12.9)	18(12)	12(8.0)	49(11.0)
Other	9(6.1)	2(1.3)	3(2.0)	14(3.1)

^a^ Parents, siblings, spouse, partner, children.

^b^ Nephews, nieces, grandchildren.

^c^ Friends, neighbors.

### Results of qualitative analysis

As discussed above, social support was divided into four types: Economic, Material, Care Assistance, and Emotional. In addition, respondents identified people from whom they received assistance in order of closeness and importance for them. First, they received help from their spouse or domestic partner; next, children and extended family, followed by friends. To a lesser degree, the respondents mentioned help from government programs, civil society organizations and religious groups. Below are described the most common dimensions of support, as well as the persons who were recognized as sources of frequent aid. (Table 5).

**Table 5:** Overview of the most important types of social support by area of residence.

	INDIGENOUS LOCALITIES			RURAL AREAS			URBAN AREAS		
Type of support									
Who supports the patient?	Economic	Emotional	Treatment assistance	Economic	Emotional	Treatment assistance	Economic	Emotional	Treatment assistance
**Wife/ spouse**		Advises on the ties to establish with the extended family to avoid family conflicts arising from the change of role due to the disease.	Prepares food according to medical recommendations.						Prepares food according to medical recommendations.
(For male patients)		Supports compliance with medical recommendations to prevent complications resulting in extra expenses to the household economy.	Accompanies the patient to exercise.		Accompanies the patient to medical appointments.	Attentive to medication and medicinal plants used to treat the disease.		Accompanies the patient to medical appointments.	Accompanies the patient to exercise.
			Restricts the intake of foods forbidden by the doctor.						Takes care of what food the patient eats at parties.
**Husband/spouse**		Advises his spouse to continue forward with her life despite the disease.						Reduces demands on domestic responsibilities of his spouse.	Scolds the patient for not complying with treatment.
(For female patients)	Pays private medical consultations and laboratory tests.	Reduces his demands on his spouse’s domestic responsibilities.		Pays private medical consultations, medications; main source of income in the household.	Becomes more sympathetic	Supports the elaboration of food that can be consumed by his spouse.	Pays private medical consultations, medications; main source of income in the household.	Excuses the absence of sexual life.	Buys home exercise equipment so that the patient does not have to exercise outdoors.
		Improves his performance as father and spouse at home.						Helps morally in times of crisis with expressions of affection.	
								Accompanies the patient to medical appointments.	
**Sons/Daughters**	Pay private medical consultations, surgeries, buy medications.	Show solidarity with the patient by not eating food the patient cannot eat.	Accompany the patient to medical appointments and laboratory studies.	Pay private medical consultations; buy medications.		Accompany the patient to medical appointments.	Pay private medical consultations and medications.	Encourage compliance with treatment.	Watch the participation of the patient in the MSG.
(For ill parents)	Buy food		Provide food (recommended by the doctor and not recommended).	Buy food.		Accompany the patient to exercise.	Contribute with cash money to the household economy.	Emotional support	Remind the patient of the time to take medications.
	Contribute with cash money to the household economy.	Adhere to the patient’s diet	Provide post-surgery care at home (bathing, dressing the patient).	Contribute with cash money to the household economy.				Behave better	Accompany the patient to medical appointments and keep track of them.
			Supervise alcohol consumption.						Watch that the patient does not eat food forbidden by the doctor.
	Perform informal economy activities to support costs of disease.		Watch what their sick parents eat.		Became more sympathetic				Provide food recommended by the doctor.
			Help a patient with physical disability by moving, feeding, or accompanying the patient to medical appointments.						Are aware of the results of medical examinations.
			Women-daughters help preparing food at home.						Perform first aid if needed.
			Accompany the patient to exercise.						
**Other family**			Remind the patient of medical appointments.	Parents support the treatment of their children; they pay private medical consultations and laboratory studies.		Accompany the patient to medical appointments.	Pay for medication		Enforce that the patient does not eat food forbidden by the physician.
(parents, siblings, grandchildren, brothers and sisters-in law, nephews and nieces)		Respondents commented that having a strong bond with the extended family helps to maintain a positive attitude toward the disease.	Act as translators for the patient.	Buy products for family consumption.	Became more sympathetic toward the behavior of the patient; they treated the patient better.	When the patient is a son or a daughter, the mother makes special food for him or her.	Buy food		Carry prepared food allowed to the patient.
			Act as readers when the patient cannot read or write.						
			Provide resources for daily life; fetch water and carry prepared food to the patient.			Help a patient with physical disability by moving or accompanying the patient to medical appointments.			
**Friends**		Listen to and chat with the patients	Accompany the patient to medical appointments.		Share moments of leisure and relaxation.	Accompany the patient to medical appointments.	Lend money without charging interest.	Home visits to listen to the patient.	Carry prepared food to the patient.
					Listen to the patient and share common interests.		In neighborhood stores obtain credit to buy food without being charged interest.		Accompany the patient to medical appointments.

Source: Data collected by authors

#### Economic support

Money represented a permanent topic in the narrative of the interviewed patients. Obtaining money to purchase medicines and laboratory studies was stressful, and for those in worse economic situations, getting the resource represented an excessive effort. Indigenous persons also had to spend money to move to towns where they were offered medical services. The patients with longer evolution of the disease and with physical complications, usually over 60 years of age, tended to emphasized these monetary problems more due to their inability to continue working and their need to rely on family to cover the costs of treatment. These patients had to wait for their partner, children, a member of the family or friends to help them economically.

Because most women from the sample were housekeepers, they usually performed informal economic activities to deal with this situation. In urban areas, women sold kitchenware for households; in rural areas, they offered different types of prepared food; in indigenous localities, they traded with plants, flowers and fruits. These strategies helped resolve the issue of the money day-to-day, but did not have a desirable impact on self-care or adherence to treatment, since these women spent all day outside the home.

Urban respondents mentioned family collective strategies to deal with the expenses, such as making use of “credits” in neighborhood stores or asking for money from family and friends.

*“…These people care about me*. *Olga will be about thirty -something*, *ma’am Mica is about sixty or so*, *her husband also cares because when he needs something and I have money he asks me to loan it to him and I do*. *And when I need to borrow money and he has it*, *he loans it to me and so on; we all get along great*…*”*

(Woman, Urban, Coahuila, lines: 250:259).

The respondents from rural areas and indigenous localities reported other family strategies: join the income of members of the extended family (sons-in-law, daughters-in-law); make purchases in common with family and neighbors, share expenses, share a house to avoid paying rent, leave the children in the care of relatives.

*“… Well when you’re poor where is the money going to come from when someone gets sick*?…*”*

(Woman, Rural, Chiapas, lines: 778:820)

Also, respondents who were receiving remittances from the United States shared them with family in order to buy medications. Given the economic precariousness of these families, a constant concern of patients was their future as chronically ill persons. Among elderly respondents there was fear that children would leave to form their own family and stop supporting them.

*“…But now he’s got a wife*, *unfortunately she came with two kids*, *and he doesn’t spend anything on me anymore*, *he gives it to the mother [the wife]…*

(Woman, Urban, Chiapas, lines: 267:284)

*“…Once you get married your wife isn’t going to want you to help me out any more…”*

(Woman, Rural, Chiapas, lines: 375:399)

They expected more economic help from sons than from daughters due to gender, as daughters could support them only if allowed by their husband. An indigenous man stated:

*“…My wife here*, *she helps me*, *she fawns over me like a baby*. *I like* pozol, *I like food*, *whatever it is she makes it for me*, *that’s why she got married*… *the husband has to ask*, *it’s the woman’s job to take care of the house*: *do the laundry*, *fold clothes…And we men work in the field growing corn…”*

(Man, Rural, Chiapas, lines: 307:328)

The variation of social resources dropped more in indigenous localities, than in rural areas and was confined to the extended family. The testimonies of indigenous respondents demonstrated the association between economic restriction and not attending medical appointments or having treatment monitored. Only government poverty alleviation programs helped to continue treatment by means of fund transfers. To that end, support from Civil Society Organizations (CSOs) was also reported, with some evidence of economic benefits distributed to families. These benefits are minimal but acquire importance in contexts of great social vulnerability. This was reported in the state of Chiapas, which has a significant percentage of indigenous population and many active OSCs.

#### Assistance with care and treatment

Assistance to address nutritional needs for treatment: This type of support depended on the gender and role of the patient within the household. In the case of female patients, the organization of domestic life became complicated due to their role as wife-mother-patient. Those with more economic resources made two types of food (non-diet and dietetic food); only a few respondents reported following that strategy. For male patients, their wives were responsible for preparing the food recommended by the doctor and watching the type and amount of food consumed. In the case of urban men with no spouse, some declared receiving this type of dietary care from their mother, even if they were independent adults. This same behavior was found in rural areas and indigenous localities, although it differed as to food preparation; in those homes usually the female patient prepared only one type of food for the whole family. The role of children was to care for the sick parent to consume only as prescribed.

Help taking medications at the right time: In all three types of residence areas, the wife monitored the taking of medications for male patients, and when the patient was a woman that task corresponded to the children.

Help performing physical activity: In urban areas the respondents were more informed and willing to follow this recommendation, but not so in other residence types. In some cases, patients were accompanied for walks or for practicing physical activity organized by the MSG.

Being accompanied to medical appointments: In all three areas of residence, children accompanied parents to medical consultations. In urban environments they encouraged the participation of their parents in MSG educational sessions. In indigenous localities, children also accompanied their parents to undergo laboratory tests. In contrast, an indigenous respondent talked about the reluctance of health personnel to accept the presence of relatives in the exam room and to give information to children about their parents’ health status,

*“…I told him*, *do not get angry at me*, *doctor*, *but my daughters ask for my sugar and my pressure and my weight and I write it down right now*, *because I forget*…*”*

(Woman, Urban, Chiapas, lines: 206:207).

Assistance for patients disabled by physical complications: In these cases, the children were the main providers of care for patients within the home, by bathing them, changing their clothes, moving them from one side to another, preparing food and feeding them, and providing postoperative care. Help was also received from other family members, such as parents or sisters- and brothers-in law. Indigenous patients were supported with translation during the medical consultation and prescription reading, and by being reminded of their medical appointments.

#### Emotional support

The assistance provided by people in their immediate environment aimed to strengthen their self-esteem, since patients developed frequent depressive episodes and feelings of sadness. Newly diagnosed patients reported experiencing stressful moments; they were afraid of their future, of developing any complications. Patients who already had complications were frustrated and their narrative revealed a life of suffering as a consequence of the disease.

Being accompanied was considered the main emotional support. It was valued as an important component of the patients’ everyday lives, especially when they had a disability that made them feel useless. In urban areas, respondents asked neighbors, friends, and close relatives to go with them to MSG sessions. Also, patients joined other ill persons to carry out, group home visits to accompany a sick neighbor. In rural areas, Catholic families took patients to church.

Role change: Due to their health condition, respondents relinquished their role as economic providers for their nuclear and extended family. In urban settings, women abandoned their role as wives; their sex life declined, but they were understood by their partner.

Decrease in domestic duties: Children adapted well to changes in daily life; they became more affectionate, showed better behavior and collaborated with the housework once they knew about their parents’ diagnosis of diabetes. Women were treated better by their husband and family demands on their domestic chores also decreased.

Counseling: In all three types of residence, friends played an important role by listening to and advising the patient, and by encouraging the patient’s perspective on carrying on with life despite having a chronic illness. Counseling allowed patients to overcome depressive episodes and forget suicidal thoughts. In indigenous localities, diabetes was viewed as a “curse”, according to their cultural beliefs. There, friends recommended treatments based on indigenous traditional medicine.

Scolding as a sign of affection: Family frequently rebuked the patient for not following medical recommendations; these reprimands were seen as a manifestation of affection. In some cases, support of local priests was requested in convincing the patient to comply with treatment. At the request of the family, priests also rebuked patients for their “bad behavior” when they did not follow medical recommendations.

#### Material aid

In the three types of localities, family and close friends contributed with food for the patients, which was pointed out by respondents as the most frequent aid they received. In urban areas this happened as a gesture of solidarity with patients who lived alone. Among the indigenous population and in rural areas this type of support was greatly valued, given the conditions of economic precariousness of patients from those areas. When questioned: *“How does your family support you*?*”* a female indigenous respondent of Chiapas said: *“By passing me a glass of water”* (Female, Villa las Rosas, Chiapas, lines: 919:945).

In indigenous localities, help from the spouse, family, and friends in various activities of daily living, such as fetching water, was greatly appreciated, especially by female patients with disabilities. One said:

*“…He* [the husband] *doesn’t bring me my food*. *What food am I going to eat*?… *He really doesn’t help me*, *and if he does that’s when things are okay*. *But he doesn’t even bring me water*, *he doesn’t even fill the buckets for me and leaves*, *and I say*: *will you fill those buckets up*, *the ones back there*: *‘yeah*, *yeah*, *’ he says*, *‘when I get back I’ll fill them’ or if not then he goes ‘I’ll come get you to have breakfast in the park where they sell stuff*.*’ Sure*, *I say*, *I wait*, *I wait*, *nothing*, *he doesn’t show up*, *I tell him my eyes could dry out and you still don’t show up*, *‘it’s that I get drunk and forget everything…'*

[he tells me] (Woman, Indigenous, Yucatan, lines: 103:109; 550:559).

Support outside the nuclear family: Government programs to reduce poverty, Christian churches, and CSO’s were tangentially mentioned by some respondents; for example, some organizations donated wheelchairs. This type of support was not homogeneous, but it was significant for the most vulnerable patients with cumulative negative life conditions: the elderly, amputees, the blind, indigenous individuals, women, and those living in poverty. These paradigmatic cases had to seek the help of these types of organizations who viewed them as destitute, socially excluded and marginalized from the possibilities of institutional care. For example, for an indigenous amputee to receive a wheelchair was more a matter of luck than an act resulting from demanding their right to health. Additionally, some respondents from rural areas mentioned being accompanied by parishioners of Christian churches to their homes for group prayer.

Reciprocity: The informants who lived alone, were widows or single mothers received less help with their disease than those with more established a network of support. Those who found themselves living in those circumstances talked about the distinct lack of support and care from people who were capable of visiting, saying they *‘had nothing to offer*.’ This assessment was directly linked to a lack of financial resources to be able to reciprocate any kind of help received from visitors. This sentiment extended even to children and close relatives.

Question: *Your children no longer live at home*?

Answer: “…*No*, *they didn’t live with me anymore*, *everyone had their own house and yes*, *they would come visit but after their father died* [they stopped coming]… *since I don’t have any money to give them*, *why would they come see me*?…*when I could give them something then of course*, *they* [her children] *would all be here*…”

(Woman, Indigenous, Chiapas, lines: 375:399).

These people said they would rather not have visitors than go through the embarrassment of not having any financial resources to return the favor. They considered that at minimum, to be reciprocal in returning the favor of the visit, one should be able to at least offer a refreshment.

#### Structure of respondents’ support network

**Fig 1:**
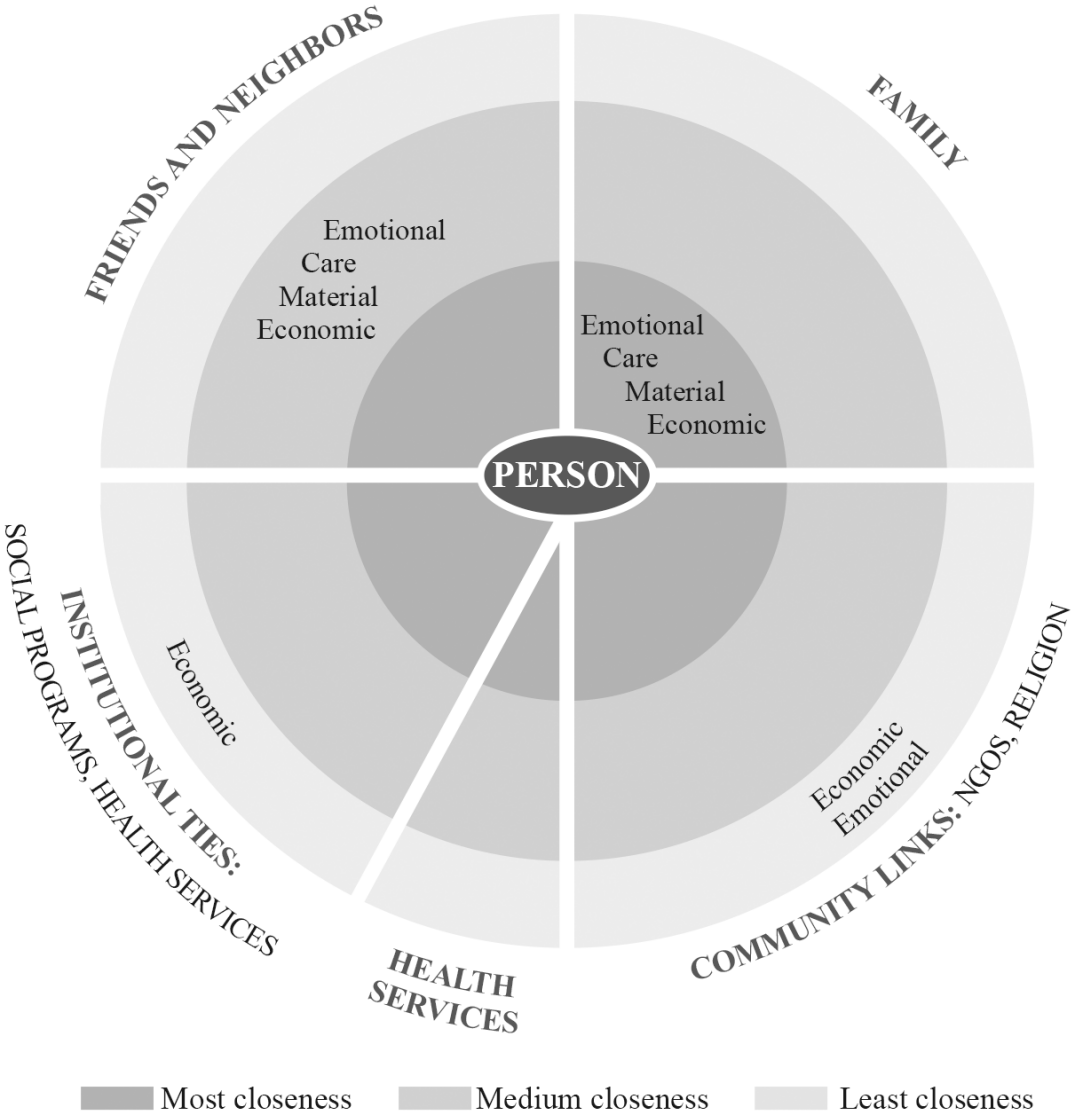
Providers of social support for patients with type 2 diabetes with economic precariousness.

Fig 1 represents an analysis of the structure of respondents’ support network and their need for social support due to their disease, according to the frequency of mention and the degree of closeness perceived by the patient, following an analysis proposal about social network from Sluski [32]. The support received by respondents came first from the nuclear family, to a lesser extent from friends, and finally from social programs. The most vulnerable cases received assistance from other community groups, such as civil society organizations and religious groups. In these cases the fabric of social network was activated to respond to four types of needs, in order of importance: economic, care support for treatment adherence; emotional, and material. Fig 1 shows a quadrant displaying institutional ties and the type of support mentioned by participants; notably, the space intended for health services is empty, because they were not mentioned. This was the case in all three types of settings where information was collected.

## Discussion

Our results suggest three aspects to discuss with grave implications both at the individual and social level regarding the problem of compliance with diabetes treatment.

### Support and social capital in precarious contexts

Our findings are representative of a population group in precarious conditions that fully depend on its social capital to deal with the disease. In this context, the cooperation received from the support network at survival levels is also precarious. This minimal support, however, is of great significance to withstand illness. This condition of life gives a different meaning to the actions of support received, which are perceived more in terms of quality than quantity [17].

The type of support was related to the gender of the respondents; support differed depending on whether the patient was male or female. Likewise, support needs were different according to the condition of the patient. If conditions are considered precarious, support is constructed on the assumption that ‘*what is received is right’*; support is thus given as a solidarity action. In the case of patients with type 2 diabetes, other authors report that having company and a well-established family and social network significantly increases the patients’ perception on their individual capacity to manage treatment [33, 34]. The company of family and friends provides a greater sense of emotional well-being and increases patients’ health empowerment; they make better decisions regarding their disease and the regulation of activities they need to perform in their immediate environment to comply with treatment, which has a positive effect on disease control. These variables carry more weight than social class or socioeconomic status [35]. Investigations on stress and depression in chronically ill persons show differences in the narrative of people who perceive to have a support network “available” to help them in comparison with those who perceive not to have it [36]. There is also evidence of the relationship between living alone and the risk of developing type 2 diabetes [37].

As for the *reciprocity* of exchange, from our findings we should discuss and reflect on what happens when patients are unable to participate in the exchange and cannot return the gesture because of economic reasons. This has an impact on the emotional life of patients; it is not just the value of the given object; returning a favor is an important part of the social construction of individuals and their values, dignity, and good reputation [38].

Social capital does not take away the responsibility of the health system to support the sick. Daly and Silver [39] have discussed the danger of this for public policies in a globalized world and in economic restructuring. Believing that the most disadvantaged population groups can survive only by their social capital can promote inequality and inequities [40].

### The role of the family in the self-management of the disease

Other studies indicate that the role of people close to patients is critical for complying with treatment and for helping dealing with stress [41]. This research, however, has identified details of daily life that hinder adherence to treatment. Close family and friends collaborate in the caring process various ways, but this does not mean that the disease is being controlled. And yet the support of the social network plays a decisive role in the development of the skills needed by the patient to self-manage the disease [42]. In the cases presented here, by providing simple aid consistent with their economic reality, the family and friends subsidize and reduce the load on the state and health services; otherwise the demand for care would surpass their ability to manage the social health system. Analyzing the social environment, Bauman considers that social relations in the 21st century are characterized by their transience [43]. Despite this, however, there is a *community*, i.e., the union of people contributing through established ties to their mutual security, as a response to the individualization and disengagement that characterize the affective attachments in modern societies. Our findings coincide in part with this assessment, since in all three types of localities investigated, the main support came from the nuclear family. The expansion of social ties and the transience of relationships, therefore, seem to be a feature of complex societies and of belonging to a social class with higher income that enables mobility. In rural areas and indigenous localities, on the other hand, the exchange of support stems from kinship relationships based on trust.

### The challenge for health systems

The lack of money to pay treatment costs is one of the main obstacles to compliance with medical recommendations. Having social and economic resources promotes self-esteem and enhances the perception of the power to make decisions in everyday life, which is related to “self-sufficiency” to follow a medical treatment, according to Ciechanowski. These investigators reported greater adherence to treatment among patients who felt self-sufficient, compared with those who did not perceive to have control of their lives as a result of living with a chronic illness [44].

The health system must guarantee the availability of medication; properly managing the services and supplies required for the treatment of diabetes is the most appropriate way to ensure that patients will continue prescribed medical treatment and to promote compliance [45, 46]. The education of family members on issues related to home care, including diet, is an area of opportunity for the diabetes care program [47]. Other authors have shown that family support is more effective than health services and programs for making lifestyle changes required for disease management [41]. The challenge for health services is apparent at different levels of care provision, although the main responsibility would lie with the first level of care, since patients establish the initial link with the health system at this level [48, 49]. As a result international health organizations insist on the need to strengthen systems to improve adherence to treatment; after all, that is where diagnosis and monitoring are done, metabolic indicators are controlled and educational activities are carried to prevent the development of new cases as well as detect them in a timely manner. Traditional models of diabetes care need to include the social determinants of health as aspects that influence adherence to treatment, and consider beliefs about illness and the emotional aspect of chronic disease. Studies about other chronic diseases have shown that strengthening mutual support at these levels of social relationships positively influences patients’ therapeutic behavior [50].

The WHO is considering adding another Millennium Development Goals objective of a 25% reduction in new cases of diabetes by 2025. However, we agree with the authors who point out the need to strengthen primary health care, using contextual population health status data for designing programs and adjusting goals to regional characteristics, which would help to reduce health inequities [51–53]. The results presented here give us tools to understand that health care delivery functions better if clinical knowledge is combined with patient participation and involvement with family in supporting him or her, as suggested by social participation programs for patients with chronic diseases in countries like Belgium [48]. Clinic staff in should consider the family as its main ally in prevention and management. Another outstanding issue is patient education; social support is critical to generate synergy between institutional care and diabetes self-management [54]. This point has double implications. First, it is important to care for to the sick, for example in the case of preparing meals, but is that adequate for the needs of the patient? Training the family on issues related to home care, including food, is an area of opportunity for diabetes care programs. Massive information campaigns to identify risk factors should also be implemented, as well as better strategies to provide aid to patients [48]. Finally, we wish to highlight the perception of lack of support from public health services as shown in Fig 1, and the contribution of this to health inequalities in terms of negative impact on the recovery of patients with low social capital. Future research should ask what kind of bias is building up within these patients and how a lack of institutional support promotes social unrest.

## Conclusions

Our findings show that in precarious economic and social conditions, the nuclear family is an integral part of the social fabric that most helps patients deal with type 2 diabetes. The main need for the patients in our sample was economic support. The lack of compliance with treatment is linked to difficulties in paying expenses derived from medical recommendations. A support network assists the patient in many ways and helps them cope with their disease but does not guarantee quality of care nor enable self-management of treatment; therefore, health services should integrate the family in diabetes care strategies and at least guarantee the supply of medications.

### Limitations of the study

In the quantitative study gender was not statistically significant, while the qualitative results highlighted how much women in the home take part in caring for the sick. This finding could represent biases about how the disease at hand is cared for and understood. Future studies should investigate the same issues with male patients and the sample should be expanded outside public health services, as there is evidence of higher use of health services by women compared with men, which could have biased the results of this study. The family can also be a negative factor for the patient if they are not sensitive to their treatment needs. Therefore future research may want to extend the inquiry to at least members of the nuclear family. Finally, data collection for this research took several years, so the health services situation may be different today than at the time we conducted our field work.

## Supporting Information

S1 Fig Code Tree all Categories. (PDF)

S2 Fig Code Tree Category IX: Social Support. (PDF)

S1 Table Dictionary Quantitative Variables (PDF)

S2 Table Quantitative Data Base, Social Support. (PDF)
