# Supplementary figures and images for: Social Support of Patients with Type 2 Diabetes in Marginalized Contexts in Mexico and Its Relation to Compliance with Treatment: A Sociocultural Approach
Source: PLoS One. 2015 Nov 6;10(11):e0141766. doi: 10.1371/journal.pone.0141766 (PMC4636160; doi:10.1371/journal.pone.0141766)

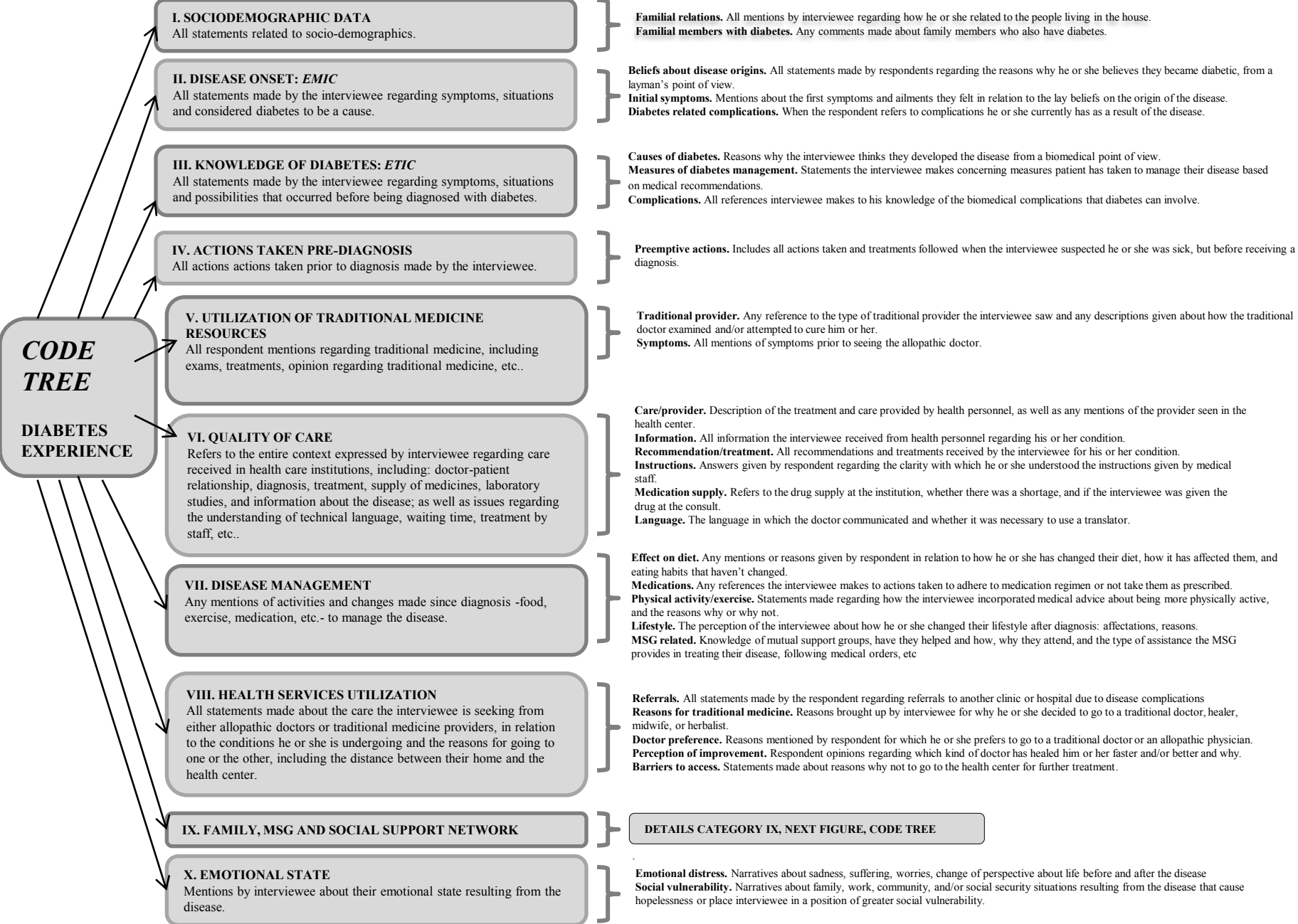

Supplement: S1 Fig — (PDF) [file pone.0141766.s001.pdf]

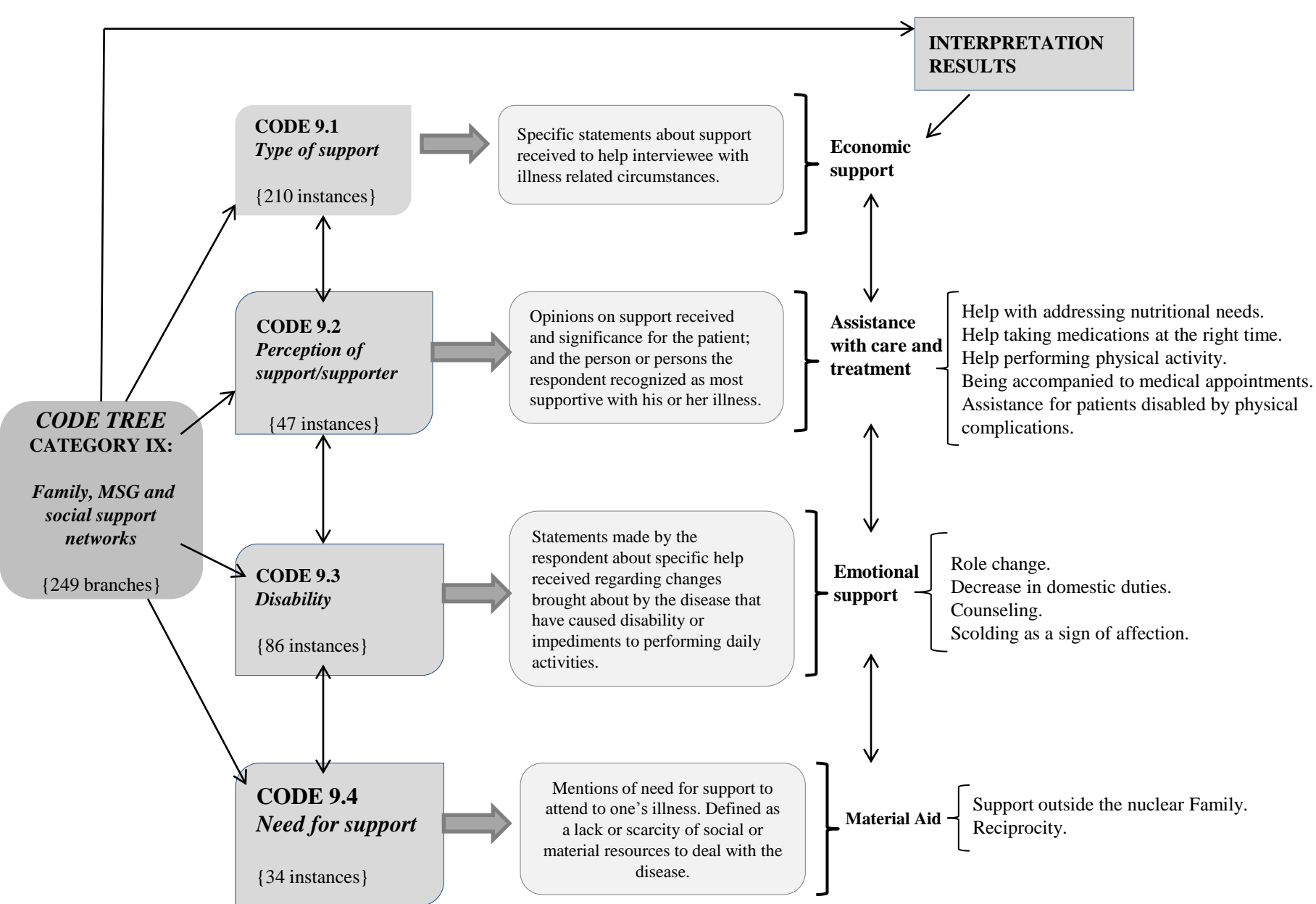

Supplement: S2 Fig — (PDF) [file pone.0141766.s002.pdf]
