# Supplementary material for: Social Support of Patients with Type 2 Diabetes in Marginalized Contexts in Mexico and Its Relation to Compliance with Treatment: A Sociocultural Approach
Source: PLoS One. 2015 Nov 6;10(11):e0141766. doi: 10.1371/journal.pone.0141766 (PMC4636160; doi:10.1371/journal.pone.0141766)
Supplement: S1 Table — (PDF) [file pone.0141766.s003.pdf]

Dictionary: Quantitative Variables

Variable name	Variable description	Coding
ID		
edadf	Age in years	Range: 21 - 86
tipoMpio	Type of locality	0=indigenous, 1=rural, 2=urban
sexo	Sex	1=Male, 2=Female
gpo_edad2	Age group	1=21-49 years, 2=50-64 years, 3= 65-86 years
edociv4	Marital status	1=Single, 2=Separated/Divorced, 3=Widowed, 4=Married/Cohabitating
school	Schooling	1=illiterate, 2=elementary, 3=higher than elementary
p77	Occupation	1=housekeeper, 2=farmer or trader, 3=worker, 4=professional or other
progrm	Beneficiary of government assistance program	1=Yes, 2=No
tdiab5	Time elapsed since diabetes diagnosis	1=3 years or fewer, 2=4-12 years, 3=more than 12 years
p31	Had complications	1=Yes, 0=No
comp_Neuro	Ulcers	1=Yes, 0=No
comp_Amp	Amputations	1=Yes, 0=No
comp_Nefro	Nephropathy	1=Yes, 0=No
comp_Vista	Visual impairment	1=Yes, 0=No
comadbt	Diabetic coma	1=Yes, 0=No
p89	Had any support	0=no support, 1=some support
Apoy_eco	Economic support	1=Yes, 0=No
Apoy_especie	Material support	1=Yes, 0=No
Apoy_cuid	Treatment support	1=Yes, 0=No
Apoy_afect	Emotional support	1=Yes, 0=No
Apoy_otro	Other support	1=Yes, 0=No
p57	Consumption of tobacco	1=Yes, 0=No
p59	Consumption of alcohol	1=Yes, 0=No
p85	Family composition	0=lives alone, 1=lives with relative
p96	Family history of diabetes	1=Yes, 0=No
conocDM	Knowledge of disease	1=Yes, 0=No
scare	Self-care	1=Yes, 0=No
p43	Satisfied with medical care	1=Yes, 2=No, 3=regular
trad2	Use of traditional medicine	1=Yes, 0=No
