# Supplementary material for: Social Support of Patients with Type 2 Diabetes in Marginalized Contexts in Mexico and Its Relation to Compliance with Treatment: A Sociocultural Approach
Source: PLoS One. 2015 Nov 6;10(11):e0141766. doi: 10.1371/journal.pone.0141766 (PMC4636160; doi:10.1371/journal.pone.0141766)
Supplement: S2 Table — (PDF) [file pone.0141766.s004.pdf]

ID	edadf	tipoMpio	sexo	gpo_edad2	edociv4	school	p77
1	52	Indigenous localities	Female	50-64 years	Married/Cohabitation	Elementary	Housekeeper
2	43	Indigenous localities	Female	21-49 years	Married/Cohabitation	Elementary	Housekeeper
3	56	Indigenous localities	Female	50-64 years	Married/Cohabitation	Elementary	Former or trader
4		Indigenous localities	Female		Married/Cohabitation	Illiteracy	Housekeeper
5	52	Indigenous localities	Female	50-64 years	Separated/Divorced	Elementary	Housekeeper
6	37	Indigenous localities	Female	21-49 years	Married/Cohabitation	Elementary	Housekeeper
7	61	Indigenous localities	Female	50-64 years	Married/Cohabitation	Illiteracy	Housekeeper
8		Indigenous localities	Female		Married/Cohabitation	Elementary	Housekeeper
9	60	Indigenous localities	Female	50-64 years	Widowed	Elementary	Housekeeper
10	65	Indigenous localities	Female	65-86 years	Separated/Divorced	Elementary	Housekeeper
11	52	Indigenous localities	Female	50-64 years	Widowed	Elementary	Housekeeper
12	51	Indigenous localities	Female	50-64 years	Married/Cohabitation	Elementary	Housekeeper
13	41	Indigenous localities	Female	21-49 years	Married/Cohabitation	Elementary	Housekeeper
14	54	Indigenous localities	Female	50-64 years	Married/Cohabitation	Elementary	Housekeeper
15	59	Indigenous localities	Female	50-64 years	Married/Cohabitation	Elementary	Former or trader
16	79	Indigenous localities	Female	65-86 years	Widowed	Elementary	Former or trader
17	52	Indigenous localities	Female	50-64 years	Single	Elementary	Housekeeper
18	54	Indigenous localities	Female	50-64 years	Married/Cohabitation	Elementary	Housekeeper
19	51	Indigenous localities	Female	50-64 years	Married/Cohabitation	Illiteracy	Housekeeper
20	44	Indigenous localities	Female	21-49 years	Married/Cohabitation	Elementary	Housekeeper
21	34	Indigenous localities	Female	21-49 years	Married/Cohabitation	Elementary	Housekeeper
22	37	Indigenous localities	Female	21-49 years	Married/Cohabitation	Elementary	Housekeeper
23	36	Indigenous localities	Female	21-49 years	Single	Higher than elementary	Housekeeper
24		Indigenous localities	Female		Married/Cohabitation	Elementary	Housekeeper
25	49	Indigenous localities	Female	21-49 years	Married/Cohabitation	Elementary	Housekeeper
26		Indigenous localities	Female		Separated/Divorced	Elementary	Housekeeper
27	59	Indigenous localities	Female	50-64 years	Married/Cohabitation	Elementary	Proffessional or other
28		Indigenous localities	Female		Single	Elementary	Housekeeper
29	47	Indigenous localities	Men	21-49 years	Married/Cohabitation	Elementary	Housekeeper

30	55	Indigenous localities	Female	50-64 years	Married/Cohabitation	Elementary	Housekeeper
31	44	Indigenous localities	Female	21-49 years	Married/Cohabitation	Elementary	Housekeeper
32	40	Indigenous localities	Female	21-49 years	Married/Cohabitation	Elementary	Housekeeper
33	49	Indigenous localities	Men	21-49 years	Married/Cohabitation	Elementary	
34	75	Indigenous localities	Female	65-86 years	Married/Cohabitation	Elementary	Housekeeper
35	52	Indigenous localities	Female	50-64 years	Single	Elementary	Housekeeper
36	28	Indigenous localities	Female	21-49 years	Married/Cohabitation	Illiteracy	Housekeeper
37	68	Indigenous localities	Female	65-86 years	Married/Cohabitation	Illiteracy	Housekeeper
38	53	Indigenous localities	Female	50-64 years	Married/Cohabitation	Illiteracy	Housekeeper
39		Indigenous localities	Female		Married/Cohabitation	Illiteracy	Housekeeper
40		Indigenous localities	Female		Married/Cohabitation	Illiteracy	Housekeeper
41	49	Indigenous localities	Female	21-49 years	Married/Cohabitation	Elementary	Housekeeper
42	51	Indigenous localities	Female	50-64 years	Married/Cohabitation	Elementary	Housekeeper
43	64	Indigenous localities	Female	50-64 years	Married/Cohabitation	Illiteracy	Former or trader
44	76	Indigenous localities	Female	65-86 years	Married/Cohabitation	Illiteracy	Former or trader
45	69	Indigenous localities	Female	65-86 years	Married/Cohabitation	Illiteracy	Housekeeper
46	77	Indigenous localities	Female	65-86 years	Married/Cohabitation	Illiteracy	Housekeeper
47	61	Indigenous localities	Female	50-64 years	Single	Elementary	Housekeeper
48	42	Indigenous localities	Men	21-49 years	Married/Cohabitation	Elementary	worker
49	54	Indigenous localities	Female	50-64 years	Married/Cohabitation	Illiteracy	Housekeeper
50		Indigenous localities	Female		Married/Cohabitation	Illiteracy	Housekeeper
51		Indigenous localities	Female		Married/Cohabitation	Elementary	Housekeeper
52		Indigenous localities	Men		Married/Cohabitation	Elementary	worker
53		Indigenous localities	Female		Married/Cohabitation	Illiteracy	Housekeeper
54		Indigenous localities	Female		Married/Cohabitation	Illiteracy	Housekeeper
55		Indigenous localities	Female		Married/Cohabitation	Elementary	Housekeeper
56	42	Indigenous localities	Female	21-49 years	Married/Cohabitation	Elementary	Housekeeper
57	65	Indigenous localities	Female	65-86 years	Married/Cohabitation	Elementary	Housekeeper
58	62	Indigenous localities	Female	50-64 years	Married/Cohabitation	Illiteracy	Housekeeper
59	53	Indigenous localities	Female	50-64 years	Married/Cohabitation	Illiteracy	Housekeeper

60	47	Indigenous localities	Female	21-49 years	Married/Cohabitation	Illiteracy	Housekeeper
61	69	Indigenous localities	Female	65-86 years	Married/Cohabitation	Illiteracy	Housekeeper
62		Indigenous localities	Female		Married/Cohabitation	Illiteracy	Former or trader
63		Indigenous localities	Female		Married/Cohabitation	Illiteracy	Former or trader
64		Indigenous localities	Female		Married/Cohabitation	Illiteracy	Housekeeper
65	62	Indigenous localities	Female	50-64 years	Married/Cohabitation	Elementary	Housekeeper
66		Indigenous localities	Female		Married/Cohabitation	Illiteracy	Housekeeper
67	49	Indigenous localities	Men	21-49 years	Married/Cohabitation	Elementary	Housekeeper
68	56	Indigenous localities	Female	50-64 years	Married/Cohabitation	Illiteracy	Housekeeper
69		Indigenous localities	Female		Widowed	Illiteracy	Housekeeper
70	53	Indigenous localities	Female	50-64 years	Married/Cohabitation	Elementary	Housekeeper
71		Indigenous localities	Female		Married/Cohabitation	Illiteracy	Housekeeper
72	64	Indigenous localities	Female	50-64 years	Married/Cohabitation	Elementary	Housekeeper
73	50	Indigenous localities	Female	50-64 years	Married/Cohabitation	Elementary	Housekeeper
74	49	Indigenous localities	Female	21-49 years		Elementary	Housekeeper
75	55	Indigenous localities	Female	50-64 years	Married/Cohabitation	Illiteracy	Housekeeper
76	34	Indigenous localities	Female	21-49 years	Separated/Divorced	Elementary	Housekeeper
77	72	Indigenous localities	Female	65-86 years	Separated/Divorced	Elementary	Housekeeper
78	39	Indigenous localities	Female	21-49 years	Married/Cohabitation	Elementary	Housekeeper
79	44	Indigenous localities	Female	21-49 years	Single	Elementary	Housekeeper
80		Indigenous localities	Female		Widowed	Illiteracy	Housekeeper
81		Indigenous localities	Female		Married/Cohabitation	Elementary	Housekeeper
82		Indigenous localities	Female		Married/Cohabitation	Elementary	Housekeeper
83	65	Indigenous localities	Female	65-86 years	Married/Cohabitation	Elementary	Housekeeper
84	61	Indigenous localities	Female	50-64 years	Widowed	Illiteracy	Housekeeper
85	72	Indigenous localities	Female	65-86 years	Married/Cohabitation	Elementary	Housekeeper
86	34	Indigenous localities	Female	21-49 years	Separated/Divorced	Elementary	Housekeeper
87	47	Indigenous localities	Female	21-49 years	Married/Cohabitation	Elementary	Housekeeper
88	46	Indigenous localities	Female	21-49 years	Married/Cohabitation	Elementary	Housekeeper
89	57	Indigenous localities	Female	50-64 years	Widowed	Illiteracy	Housekeeper

90	52	Indigenous localities	Female	50-64 years	Married/Cohabitation	Illiteracy	Housekeeper
91	63	Indigenous localities	Female	50-64 years	Married/Cohabitation	Illiteracy	Housekeeper
92	55	Indigenous localities	Female	50-64 years	Married/Cohabitation	Illiteracy	Housekeeper
93	44	Indigenous localities	Female	21-49 years	Married/Cohabitation	Illiteracy	Housekeeper
94	55	Indigenous localities	Female	50-64 years	Married/Cohabitation	Illiteracy	Housekeeper
95	69	Indigenous localities	Female	65-86 years	Widowed	Illiteracy	Housekeeper
96		Indigenous localities	Female		Widowed	Elementary	Housekeeper
97		Indigenous localities	Female		Married/Cohabitation	Illiteracy	Housekeeper
98	50	Indigenous localities	Men	50-64 years	Separated/Divorced	Elementary	worker
99	51	Indigenous localities	Female	50-64 years	Widowed	Elementary	Housekeeper
100	59	Indigenous localities	Female	50-64 years	Married/Cohabitation	Elementary	Housekeeper
101		Indigenous localities	Female		Married/Cohabitation	Illiteracy	Housekeeper
102		Indigenous localities	Female		Married/Cohabitation	Illiteracy	Housekeeper
103		Indigenous localities	Female		Married/Cohabitation	Illiteracy	
104	66	Indigenous localities	Men	65-86 years	Married/Cohabitation	Elementary	Former or trader
105	50	Indigenous localities	Female	50-64 years	Married/Cohabitation	Illiteracy	Housekeeper
106	58	Indigenous localities	Men	50-64 years	Married/Cohabitation	Elementary	Housekeeper
107	42	Urban area	Female	21-49 years	Married/Cohabitation	Elementary	Housekeeper
108	71	Urban area	Female	65-86 years	Widowed	Elementary	Housekeeper
109	56	Indigenous localities	Female	50-64 years	Widowed	Illiteracy	Housekeeper
110	54	Indigenous localities	Men	50-64 years	Married/Cohabitation	Elementary	Former or trader
111	43	Indigenous localities	Female	21-49 years	Married/Cohabitation	Illiteracy	
112	62	Indigenous localities	Female	50-64 years	Single	Illiteracy	Housekeeper
113	41	Indigenous localities	Female	21-49 years	Married/Cohabitation	Elementary	Housekeeper
114	47	Indigenous localities	Men	21-49 years	Married/Cohabitation	Elementary	Former or trader
115	42	Indigenous localities	Female	21-49 years	Separated/Divorced	Illiteracy	worker
116	48	Indigenous localities	Female	21-49 years	Separated/Divorced	Illiteracy	Housekeeper
117	52	Indigenous localities	Female	50-64 years	Separated/Divorced	Elementary	Housekeeper
118	66	Indigenous localities	Men	65-86 years	Married/Cohabitation	Elementary	Former or trader
119	60	Indigenous localities	Female	50-64 years	Married/Cohabitation	Illiteracy	Housekeeper

120	68	Indigenous localities	Female	65-86 years	Widowed	Illiteracy	Housekeeper
121	62	Indigenous localities	Female	50-64 years	Married/Cohabitation	Illiteracy	Housekeeper
122	64	Indigenous localities	Female	50-64 years	Widowed	Illiteracy	Housekeeper
123	67	Indigenous localities	Female	65-86 years	Married/Cohabitation	Illiteracy	Housekeeper
124	53	Indigenous localities	Female	50-64 years	Widowed	Elementary	Housekeeper
125	73	Indigenous localities	Female	65-86 years	Married/Cohabitation	Illiteracy	Housekeeper
126	66	Indigenous localities	Female	65-86 years	Widowed	Illiteracy	Housekeeper
127	51	Indigenous localities	Female	50-64 years	Married/Cohabitation	Illiteracy	Housekeeper
128	65	Indigenous localities	Female	65-86 years	Widowed	Illiteracy	Housekeeper
129	44	Indigenous localities	Female	21-49 years	Married/Cohabitation	Illiteracy	Housekeeper
130	41	Indigenous localities	Female	21-49 years	Married/Cohabitation	Illiteracy	Housekeeper
131	53	Indigenous localities	Female	50-64 years	Married/Cohabitation	Illiteracy	Housekeeper
132	56	Indigenous localities	Female	50-64 years	Married/Cohabitation	Elementary	Housekeeper
133	48	Indigenous localities	Female	21-49 years	Separated/Divorced	Elementary	Proffessional or other
134	76	Indigenous localities	Female	65-86 years	Widowed	Illiteracy	Housekeeper
135	56	Indigenous localities	Men	50-64 years	Married/Cohabitation	Elementary	Former or trader
136	64	Indigenous localities	Female	50-64 years	Married/Cohabitation	Illiteracy	Housekeeper
137	48	Indigenous localities	Men	21-49 years	Married/Cohabitation	Elementary	Proffessional or other
138	56	Indigenous localities	Female	50-64 years	Widowed	Elementary	Housekeeper
139	63	Indigenous localities	Men	50-64 years	Widowed	Elementary	Former or trader
140	58	Indigenous localities	Female	50-64 years	Married/Cohabitation	Illiteracy	Housekeeper
141	62	Indigenous localities	Female	50-64 years	Single	Elementary	Former or trader
142	59	Indigenous localities	Female	50-64 years	Married/Cohabitation	Elementary	Housekeeper
143	58	Indigenous localities	Female	50-64 years	Married/Cohabitation	Illiteracy	Housekeeper
144	52	Indigenous localities	Female	50-64 years	Separated/Divorced	Elementary	Housekeeper
145		Indigenous localities	Female		Married/Cohabitation	Illiteracy	Housekeeper
146	36	Indigenous localities	Female	21-49 years	Single	Illiteracy	Housekeeper
147	44	Indigenous localities	Female	21-49 years	Married/Cohabitation	Elementary	Housekeeper
148	37	Indigenous localities	Female	21-49 years	Single	Elementary	Housekeeper
149	59	Indigenous localities	Female	50-64 years	Married/Cohabitation	Elementary	Housekeeper

150	62	Indigenous localities	Female	50-64 years	Married/Cohabitation	Illiteracy	Housekeeper
151	71	Indigenous localities	Female	65-86 years	Married/Cohabitation	Illiteracy	Housekeeper
152	63	Indigenous localities	Female	50-64 years	Married/Cohabitation	Illiteracy	Housekeeper
153	66	Indigenous localities	Female	65-86 years	Widowed	Illiteracy	Former or trader
154	50	Indigenous localities	Female	50-64 years	Separated/Divorced	Elementary	Housekeeper
155	42	Indigenous localities	Female	21-49 years	Married/Cohabitation	Elementary	Former or trader
156	56	Indigenous localities	Female	50-64 years	Married/Cohabitation	Illiteracy	Housekeeper
157	48	Indigenous localities	Female	21-49 years	Separated/Divorced	Elementary	worker
158	47	Urban area	Female	21-49 years	Married/Cohabitation	Higher than elementary	Proffessional or other
159	72	Urban area	Men	65-86 years	Married/Cohabitation	Elementary	Housekeeper
160	67	Urban area	Female	65-86 years	Separated/Divorced	Elementary	Housekeeper
161	79	Urban area	Female	65-86 years	Widowed	Elementary	Housekeeper
162	68	Urban area	Female	65-86 years	Married/Cohabitation	Elementary	Housekeeper
163	70	Urban area	Female	65-86 years	Widowed	Elementary	Housekeeper
164	59	Urban area	Female	50-64 years	Separated/Divorced	Higher than elementary	Proffessional or other
165	81	Urban area	Female	65-86 years	Widowed	Higher than elementary	Housekeeper
166	69	Urban area	Female	65-86 years	Married/Cohabitation	Elementary	Housekeeper
167	65	Urban area	Men	65-86 years	Married/Cohabitation	Elementary	Housekeeper
168	68	Urban area	Men	65-86 years	Married/Cohabitation	Illiteracy	Proffessional or other
169	75	Urban area	Female	65-86 years	Married/Cohabitation	Higher than elementary	Housekeeper
170	54	Urban area	Female	50-64 years	Married/Cohabitation	Elementary	Proffessional or other
171	63	Urban area	Female	50-64 years	Widowed	Higher than elementary	Proffessional or other
172	45	Urban area	Men	21-49 years	Married/Cohabitation	Higher than elementary	Proffessional or other
173	55	Urban area	Men	50-64 years	Married/Cohabitation	Elementary	Proffessional or other
174	63	Urban area	Female	50-64 years	Married/Cohabitation	Illiteracy	Housekeeper
175	51	Urban area	Men	50-64 years	Married/Cohabitation	Higher than elementary	Proffessional or other
176	57	Urban area	Female	50-64 years	Married/Cohabitation	Elementary	Housekeeper
177	39	Urban area	Female	21-49 years	Married/Cohabitation	Higher than elementary	Proffessional or other
178	65	Urban area	Female	65-86 years	Married/Cohabitation	Elementary	Housekeeper
179		Urban area	Female		Married/Cohabitation	Elementary	Housekeeper

180	44	Urban area	Men	21-49 years	Married/Cohabitation	Higher than elementary	Proffessional or other
181	79	Urban area	Female	65-86 years	Widowed	Elementary	Housekeeper
182	44	Urban area	Female	21-49 years	Married/Cohabitation	Higher than elementary	Proffessional or other
183	55	Urban area	Female	50-64 years	Married/Cohabitation	Elementary	Housekeeper
184	56	Urban area	Men	50-64 years	Married/Cohabitation	Elementary	Housekeeper
185	67	Urban area	Men	65-86 years	Married/Cohabitation	Elementary	Former or trader
186	41	Urban area	Female	21-49 years	Married/Cohabitation	Higher than elementary	Housekeeper
187	50	Urban area	Female	50-64 years	Married/Cohabitation	Higher than elementary	Proffessional or other
188	63	Urban area	Female	50-64 years	Separated/Divorced	Illiteracy	Housekeeper
189	79	Urban area	Men	65-86 years	Married/Cohabitation	Elementary	Housekeeper
190	45	Urban area	Female	21-49 years	Married/Cohabitation	Higher than elementary	Proffessional or other
191	56	Urban area	Men	50-64 years	Separated/Divorced	Elementary	Proffessional or other
192	51	Urban area	Female	50-64 years	Separated/Divorced	Higher than elementary	Proffessional or other
193	66	Urban area	Men	65-86 years	Married/Cohabitation	Higher than elementary	Former or trader
194	56	Urban area	Female	50-64 years	Married/Cohabitation	Elementary	Housekeeper
195	49	Urban area	Female	21-49 years	Separated/Divorced	Elementary	Housekeeper
196	67	Urban area	Female	65-86 years	Married/Cohabitation	Elementary	Housekeeper
197	80	Urban area	Female	65-86 years	Widowed	Illiteracy	Housekeeper
198	53	Urban area	Female	50-64 years	Married/Cohabitation	Higher than elementary	Proffessional or other
199	54	Urban area	Men	50-64 years	Married/Cohabitation	Higher than elementary	Proffessional or other
200	73	Urban area	Men	65-86 years	Married/Cohabitation	Higher than elementary	Proffessional or other
201	50	Urban area	Men	50-64 years	Married/Cohabitation	Illiteracy	Former or trader
202	40	Urban area	Female	21-49 years	Married/Cohabitation	Elementary	Former or trader
203	71	Urban area	Female	65-86 years	Widowed	Elementary	Housekeeper
204	69	Urban area	Men	65-86 years	Married/Cohabitation	Higher than elementary	Proffessional or other
205	64	Urban area	Female	50-64 years	Married/Cohabitation	Elementary	Former or trader
206	54	Urban area	Female	50-64 years	Separated/Divorced	Elementary	Housekeeper
207	72	Urban area	Female	65-86 years	Single	Illiteracy	Housekeeper
208	78	Urban area	Female	65-86 years	Widowed	Elementary	Housekeeper
209	69	Indigenous localities	Female	65-86 years	Married/Cohabitation	Elementary	Housekeeper

210	66	Indigenous localities	Female	65-86 years	Separated/Divorced	Elementary	Housekeeper
211	57	Indigenous localities	Men	50-64 years	Married/Cohabitation	Higher than elementary	Proffessional or other
212	52	Indigenous localities	Men	50-64 years	Married/Cohabitation	Higher than elementary	Proffessional or other
213	51	Indigenous localities	Female	50-64 years	Married/Cohabitation	Higher than elementary	Proffessional or other
214	57	Indigenous localities	Female	50-64 years	Married/Cohabitation	Elementary	Former or trader
215	71	Indigenous localities	Female	65-86 years	Married/Cohabitation	Elementary	Housekeeper
216	52	Indigenous localities	Female	50-64 years	Married/Cohabitation	Elementary	Housekeeper
217	42	Indigenous localities	Female	21-49 years	Married/Cohabitation	Higher than elementary	Housekeeper
218	60	Indigenous localities	Female	50-64 years	Married/Cohabitation	Elementary	Housekeeper
219	70	Indigenous localities	Men	65-86 years	Married/Cohabitation	Higher than elementary	Former or trader
220	65	Indigenous localities	Female	65-86 years	Married/Cohabitation	Illiteracy	Housekeeper
221	72	Indigenous localities	Female	65-86 years	Married/Cohabitation	Illiteracy	Housekeeper
222	59	Indigenous localities	Female	50-64 years	Married/Cohabitation	Elementary	Housekeeper
223	71	Indigenous localities	Men	65-86 years	Married/Cohabitation	Elementary	Housekeeper
224	86	Indigenous localities	Female	65-86 years	Married/Cohabitation	Illiteracy	Housekeeper
225	68	Indigenous localities	Men	65-86 years	Married/Cohabitation	Elementary	
226	67	Indigenous localities	Female	65-86 years	Married/Cohabitation	Elementary	Housekeeper
227	62	Indigenous localities	Men	50-64 years	Married/Cohabitation	Elementary	
228	63	Indigenous localities	Men	50-64 years	Separated/Divorced	Elementary	Former or trader
229	50	Indigenous localities	Female	50-64 years	Married/Cohabitation	Elementary	Housekeeper
230	68	Indigenous localities	Female	65-86 years	Widowed	Illiteracy	Former or trader
231	55	Indigenous localities	Men	50-64 years	Separated/Divorced	Higher than elementary	Proffessional or other
232	79	Indigenous localities	Men	65-86 years	Married/Cohabitation	Higher than elementary	Proffessional or other
233	50	Indigenous localities	Men	50-64 years	Married/Cohabitation	Elementary	worker
234	67	Indigenous localities	Female	65-86 years	Widowed	Elementary	Housekeeper
235	50	Indigenous localities	Female	50-64 years	Married/Cohabitation	Elementary	Housekeeper
236	80	Indigenous localities	Men	65-86 years	Married/Cohabitation	Elementary	Housekeeper
237	60	Indigenous localities	Men	50-64 years	Married/Cohabitation	Elementary	Proffessional or other
238	63	Indigenous localities	Female	50-64 years	Separated/Divorced	Illiteracy	worker
239	60	Indigenous localities	Female	50-64 years	Single	Higher than elementary	Housekeeper

240	41	Indigenous localities	Female	21-49 years	Married/Cohabitation	Elementary	Housekeeper
241	56	Indigenous localities	Men	50-64 years	Married/Cohabitation	Higher than elementary	Proffessional or other
242	61	Indigenous localities	Female	50-64 years	Married/Cohabitation	Elementary	Housekeeper
243	57	Indigenous localities	Female	50-64 years	Married/Cohabitation	Elementary	Housekeeper
244	72	Indigenous localities	Female	65-86 years	Widowed	Higher than elementary	Proffessional or other
245	65	Indigenous localities	Female	65-86 years	Married/Cohabitation	Elementary	Former or trader
246	81	Indigenous localities	Female	65-86 years	Married/Cohabitation	Illiteracy	Proffessional or other
247	74	Indigenous localities	Female	65-86 years	Married/Cohabitation	Elementary	Housekeeper
248	60	Indigenous localities	Female	50-64 years	Married/Cohabitation	Higher than elementary	Former or trader
249	54	Rural area	Female	50-64 years	Married/Cohabitation	Elementary	Housekeeper
250	66	Rural area	Female	65-86 years	Married/Cohabitation	Elementary	Housekeeper
251	61	Rural area	Female	50-64 years	Married/Cohabitation	Elementary	Housekeeper
252	63	Rural area	Female	50-64 years	Married/Cohabitation	Elementary	Housekeeper
253	37	Rural area	Female	21-49 years	Married/Cohabitation	Elementary	Housekeeper
254	64	Rural area	Female	50-64 years	Married/Cohabitation	Elementary	Housekeeper
255	66	Rural area	Female	65-86 years	Widowed	Illiteracy	Housekeeper
256	56	Rural area	Men	50-64 years	Married/Cohabitation	Elementary	worker
257	75	Rural area	Female	65-86 years	Widowed	Elementary	Housekeeper
258	62	Rural area	Female	50-64 years	Widowed	Elementary	Housekeeper
259	44	Rural area	Female	21-49 years	Married/Cohabitation	Elementary	Housekeeper
260	67	Rural area	Female	65-86 years	Widowed	Elementary	Housekeeper
261	62	Rural area	Men	50-64 years	Married/Cohabitation	Elementary	Former or trader
262	63	Rural area	Female	50-64 years	Single	Elementary	Former or trader
263	69	Rural area	Female	65-86 years	Widowed	Elementary	Housekeeper
264	61	Rural area	Men	50-64 years	Married/Cohabitation	Elementary	Former or trader
265	69	Rural area	Female	65-86 years	Married/Cohabitation	Illiteracy	Housekeeper
266	47	Rural area	Female	21-49 years	Widowed	Elementary	Housekeeper
267	64	Rural area	Female	50-64 years	Married/Cohabitation	Illiteracy	Housekeeper
268	59	Rural area	Female	50-64 years	Married/Cohabitation	Elementary	Housekeeper
269	61	Rural area	Female	50-64 years	Widowed	Elementary	Housekeeper

270	69	Rural area	Female	65-86 years	Widowed	Illiteracy	Housekeeper
271	64	Rural area	Female	50-64 years	Married/Cohabitation	Elementary	Housekeeper
272	44	Rural area	Female	21-49 years	Single	Elementary	Proffessional or other
273	48	Rural area	Female	21-49 years	Married/Cohabitation	Illiteracy	Housekeeper
274	64	Rural area	Female	50-64 years	Widowed	Elementary	Housekeeper
275	50	Rural area	Female	50-64 years	Married/Cohabitation	Elementary	Housekeeper
276	59	Urban area	Men	50-64 years	Married/Cohabitation	Elementary	Proffessional or other
277	60	Urban area	Female	50-64 years	Married/Cohabitation	Elementary	Housekeeper
278	48	Urban area	Female	21-49 years	Single	Elementary	Housekeeper
279	53	Urban area	Men	50-64 years	Married/Cohabitation	Elementary	Former or trader
280	60	Urban area	Men	50-64 years	Single	Elementary	Housekeeper
281	66	Rural area	Female	65-86 years	Married/Cohabitation	Illiteracy	Housekeeper
282	70	Rural area	Men	65-86 years	Married/Cohabitation	Elementary	Former or trader
283	52	Rural area	Female	50-64 years	Married/Cohabitation	Elementary	Housekeeper
284	65	Rural area	Female	65-86 years	Married/Cohabitation	Elementary	Housekeeper
285	69	Rural area	Female	65-86 years	Married/Cohabitation	Elementary	Housekeeper
286	53	Rural area	Female	50-64 years	Married/Cohabitation	Illiteracy	Housekeeper
287	55	Rural area	Female	50-64 years	Married/Cohabitation	Elementary	Housekeeper
288	60	Rural area	Men	50-64 years	Married/Cohabitation	Elementary	Proffessional or other
289	49	Urban area	Female	21-49 years	Married/Cohabitation	Illiteracy	Housekeeper
290	60	Urban area	Female	50-64 years	Married/Cohabitation	Elementary	Housekeeper
291	40	Urban area	Men	21-49 years	Married/Cohabitation	Elementary	Former or trader
292	59	Urban area	Men	50-64 years	Married/Cohabitation	Elementary	Proffessional or other
293	63	Urban area	Female	50-64 years	Widowed	Illiteracy	Housekeeper
294	48	Rural area	Female	21-49 years	Married/Cohabitation	Elementary	Housekeeper
295	64	Urban area	Female	50-64 years	Widowed	Illiteracy	Housekeeper
296	47	Urban area	Men	21-49 years	Married/Cohabitation	Elementary	Housekeeper
297	61	Rural area	Female	50-64 years	Married/Cohabitation	Illiteracy	Housekeeper
298	55	Urban area	Men	50-64 years	Married/Cohabitation	Elementary	Former or trader
299		Urban area	Female				Housekeeper

300	71	Urban area	Female	65-86 years	Widowed	Elementary	Housekeeper
301	64	Urban area	Female	50-64 years	Widowed	Illiteracy	Housekeeper
302	43	Urban area	Female	21-49 years	Married/Cohabitation	Elementary	Housekeeper
303	62	Urban area	Men	50-64 years	Married/Cohabitation	Elementary	Proffessional or other
304	66	Urban area	Men	65-86 years	Married/Cohabitation	Illiteracy	Former or trader
305	68	Urban area	Female	65-86 years	Married/Cohabitation	Illiteracy	Housekeeper
306	68	Urban area	Female	65-86 years	Widowed	Elementary	Housekeeper
307	61	Urban area	Female	50-64 years	Married/Cohabitation	Elementary	Housekeeper
308	63	Urban area	Men	50-64 years	Married/Cohabitation	Elementary	Housekeeper
309	56	Urban area	Female	50-64 years	Married/Cohabitation	Elementary	Housekeeper
310	50	Urban area	Female	50-64 years	Married/Cohabitation	Elementary	Housekeeper
311	55	Urban area	Female	50-64 years	Married/Cohabitation	Elementary	Housekeeper
312	62	Urban area	Men	50-64 years	Single	Elementary	Proffessional or other
313		Urban area	Men		Single	Elementary	Proffessional or other
314	69	Urban area	Men	65-86 years	Married/Cohabitation	Elementary	Proffessional or other
315	48	Urban area	Female	21-49 years	Married/Cohabitation	Elementary	Housekeeper
316	52	Urban area	Female	50-64 years	Married/Cohabitation	Elementary	Housekeeper
317	59	Urban area	Men	50-64 years	Married/Cohabitation	Elementary	Former or trader
318	54	Urban area	Female	50-64 years	Married/Cohabitation	Illiteracy	Housekeeper
319	66	Urban area	Men	65-86 years	Married/Cohabitation	Elementary	Proffessional or other
320	45	Urban area	Female	21-49 years	Married/Cohabitation	Elementary	Housekeeper
321	38	Urban area	Female	21-49 years	Married/Cohabitation	Elementary	Housekeeper
322	67	Urban area	Female	65-86 years	Married/Cohabitation	Elementary	Housekeeper
323	58	Urban area	Female	50-64 years	Married/Cohabitation	Elementary	Housekeeper
324	63	Urban area	Female	50-64 years	Widowed	Elementary	Proffessional or other
325	45	Urban area	Female	21-49 years	Single	Elementary	Housekeeper
326	69	Urban area	Men	65-86 years	Married/Cohabitation	Elementary	Former or trader
327	66	Urban area	Female	65-86 years	Married/Cohabitation	Elementary	Housekeeper
328	59	Urban area	Female	50-64 years	Married/Cohabitation	Elementary	Housekeeper
329	53	Urban area	Female	50-64 years	Married/Cohabitation	Elementary	Housekeeper

330	45	Urban area	Female	21-49 years	Married/Cohabitation	Elementary	Housekeeper
331	59	Urban area	Female	50-64 years	Married/Cohabitation	Elementary	Housekeeper
332		Urban area	Female		Married/Cohabitation	Elementary	Housekeeper
333	61	Urban area	Female	50-64 years	Widowed	Elementary	Housekeeper
334	43	Urban area	Female	21-49 years	Married/Cohabitation	Elementary	Housekeeper
335	48	Urban area	Female	21-49 years	Married/Cohabitation	Elementary	Housekeeper
336	53	Urban area	Female	50-64 years	Married/Cohabitation	Elementary	Housekeeper
337	47	Urban area	Men	21-49 years	Married/Cohabitation	Elementary	worker
338	46	Urban area	Female	21-49 years	Married/Cohabitation	Elementary	Housekeeper
339	59	Urban area	Female	50-64 years	Married/Cohabitation	Elementary	Housekeeper
340	46	Urban area	Female	21-49 years	Married/Cohabitation	Elementary	
341	56	Urban area	Female	50-64 years	Separated/Divorced	Elementary	Housekeeper
342	47	Urban area	Female	21-49 years	Married/Cohabitation	Elementary	Housekeeper
343	44	Urban area	Female	21-49 years	Married/Cohabitation	Elementary	Housekeeper
344	62	Urban area	Female	50-64 years	Single	Illiteracy	Housekeeper
345	51	Urban area	Men	50-64 years	Married/Cohabitation	Elementary	Proffessional or other
346	53	Urban area	Men	50-64 years	Single	Elementary	
347	30	Urban area	Female	21-49 years	Married/Cohabitation	Elementary	Housekeeper
348	60	Urban area	Female	50-64 years	Married/Cohabitation	Illiteracy	Housekeeper
349	68	Urban area	Female	65-86 years	Single	Elementary	Housekeeper
350	52	Urban area	Female	50-64 years	Married/Cohabitation	Elementary	Housekeeper
351	44	Urban area	Female	21-49 years	Married/Cohabitation	Higher than elementary	Housekeeper
352	53	Urban area	Female	50-64 years	Married/Cohabitation	Higher than elementary	Housekeeper
353	52	Urban area	Female	50-64 years	Married/Cohabitation	Elementary	Housekeeper
354	63	Urban area	Female	50-64 years	Married/Cohabitation	Elementary	Housekeeper
355	32	Urban area	Female	21-49 years	Married/Cohabitation	Elementary	Housekeeper
356	47	Urban area	Female	21-49 years	Married/Cohabitation	Elementary	Housekeeper
357	54	Urban area	Female	50-64 years	Separated/Divorced	Elementary	Housekeeper
358	48	Urban area	Female	21-49 years	Single	Elementary	Housekeeper
359	66	Urban area	Female	65-86 years	Widowed	Elementary	Housekeeper

360	50	Urban area	Female	50-64 years	Separated/Divorced	Elementary	Housekeeper
361	54	Urban area	Female	50-64 years	Married/Cohabitation	Elementary	Housekeeper
362	60	Urban area	Female	50-64 years	Married/Cohabitation	Elementary	Housekeeper
363	69	Urban area	Female	65-86 years	Married/Cohabitation	Elementary	Housekeeper
364	45	Urban area	Female	21-49 years	Married/Cohabitation	Elementary	Housekeeper
365	67	Urban area	Female	65-86 years	Widowed	Elementary	Housekeeper
366	68	Urban area	Female	65-86 years	Widowed	Elementary	Housekeeper
367	44	Urban area	Female	21-49 years	Married/Cohabitation	Elementary	Housekeeper
368	69	Urban area	Female	65-86 years	Widowed	Elementary	Housekeeper
369	60	Urban area	Female	50-64 years	Married/Cohabitation	Elementary	Housekeeper
370	66	Urban area	Female	65-86 years	Married/Cohabitation	Elementary	Former or trader
371	64	Urban area	Female	50-64 years	Separated/Divorced	Elementary	Former or trader
372	68	Urban area	Men	65-86 years	Married/Cohabitation	Higher than elementary	Housekeeper
373	40	Urban area	Female	21-49 years	Married/Cohabitation	Elementary	Housekeeper
374	50	Urban area	Men	50-64 years	Married/Cohabitation	Higher than elementary	Former or trader
375	47	Urban area	Female	21-49 years	Married/Cohabitation	Elementary	Former or trader
376	40	Urban area	Female	21-49 years	Married/Cohabitation	Elementary	Housekeeper
377	52	Urban area	Female	50-64 years	Married/Cohabitation	Elementary	Housekeeper
378	46	Urban area	Female	21-49 years	Married/Cohabitation	Elementary	Housekeeper
379	56	Urban area	Female	50-64 years	Married/Cohabitation	Elementary	Housekeeper
380	54	Urban area	Men	50-64 years	Married/Cohabitation	Elementary	Proffessional or other
381	64	Urban area	Men	50-64 years	Married/Cohabitation	Elementary	Proffessional or other
382	38	Urban area	Female	21-49 years	Married/Cohabitation	Elementary	Housekeeper
383	45	Urban area	Female	21-49 years	Married/Cohabitation	Elementary	Housekeeper
384	42	Urban area	Female	21-49 years	Married/Cohabitation	Elementary	Housekeeper
385	64	Urban area	Men	50-64 years	Married/Cohabitation	Elementary	Former or trader
386	69	Urban area	Female	65-86 years	Married/Cohabitation	Elementary	Housekeeper
387	64	Urban area	Female	50-64 years	Separated/Divorced	Elementary	Housekeeper
388	65	Urban area	Female	65-86 years	Married/Cohabitation	Elementary	Housekeeper
389	55	Urban area	Female	50-64 years	Separated/Divorced	Higher than elementary	Housekeeper

390	44	Urban area	Female	21-49 years	Married/Cohabitation	Elementary	Housekeeper
391	56	Urban area	Female	50-64 years	Married/Cohabitation	Elementary	Former or trader
392	67	Urban area	Female	65-86 years	Married/Cohabitation	Elementary	Housekeeper
393	40	Urban area	Female	21-49 years	Married/Cohabitation	Elementary	Housekeeper
394	67	Urban area	Female	65-86 years	Widowed	Elementary	Housekeeper
395	54	Urban area	Female	50-64 years	Married/Cohabitation	Elementary	Housekeeper
396	65	Urban area	Female	65-86 years	Separated/Divorced	Elementary	Housekeeper
397	45	Urban area	Female	21-49 years	Married/Cohabitation	Elementary	Housekeeper
398	63	Urban area	Female	50-64 years	Widowed	Elementary	Housekeeper
399	45	Urban area	Female	21-49 years	Married/Cohabitation	Elementary	Housekeeper
400	52	Urban area	Men	50-64 years	Married/Cohabitation	Elementary	Proffessional or other
401	58	Urban area	Men	50-64 years	Married/Cohabitation	Higher than elementary	Former or trader
402	56	Urban area	Female	50-64 years	Widowed	Elementary	Housekeeper
403	46	Urban area	Female	21-49 years	Married/Cohabitation	Elementary	Housekeeper
404	64	Urban area	Men	50-64 years	Married/Cohabitation	Elementary	Proffessional or other
405	64	Urban area	Female	50-64 years	Widowed	Illiteracy	Housekeeper
406	62	Urban area	Female	50-64 years	Married/Cohabitation	Higher than elementary	Former or trader
407	50	Urban area	Female	50-64 years	Married/Cohabitation	Elementary	Housekeeper
408	63	Urban area	Female	50-64 years	Single	Elementary	Housekeeper
409	50	Urban area	Female	50-64 years	Married/Cohabitation	Elementary	Housekeeper
410	67	Urban area	Men	65-86 years	Married/Cohabitation	Elementary	Proffessional or other
411	49	Rural area	Female	21-49 years	Married/Cohabitation	Illiteracy	Housekeeper
412	33	Urban area	Female	21-49 years	Married/Cohabitation	Elementary	Housekeeper
413	59	Urban area	Men	50-64 years	Single	Higher than elementary	Proffessional or other
414	65	Urban area	Female	65-86 years	Widowed	Elementary	Housekeeper
415	21	Urban area	Men	21-49 years	Single	Higher than elementary	
416	47	Urban area	Men	21-49 years	Married/Cohabitation	Elementary	Proffessional or other
417	59	Urban area	Female	50-64 years	Married/Cohabitation	Elementary	Housekeeper
418	62	Urban area	Men	50-64 years	Married/Cohabitation	Elementary	Proffessional or other
419	45	Urban area	Men	21-49 years	Single	Elementary	worker

420	52	Urban area	Female	50-64 years	Married/Cohabitation	Elementary	Housekeeper
421	68	Urban area	Female	65-86 years	Married/Cohabitation	Elementary	Housekeeper
422	51	Urban area	Men	50-64 years	Married/Cohabitation	Elementary	Proffessional or other
423	65	Urban area	Female	65-86 years	Married/Cohabitation	Elementary	Housekeeper
424	56	Rural area	Female	50-64 years	Married/Cohabitation	Illiteracy	Housekeeper
425	82	Rural area	Men	65-86 years	Married/Cohabitation	Illiteracy	worker
426	50	Rural area	Female	50-64 years	Married/Cohabitation	Illiteracy	Housekeeper
427	66	Rural area	Female	65-86 years	Single	Illiteracy	Housekeeper
428		Rural area	Female		Single	Illiteracy	Housekeeper
429	75	Rural area	Female	65-86 years	Widowed	Illiteracy	Housekeeper
430	68	Rural area	Female	65-86 years	Widowed	Illiteracy	worker
431	70	Rural area	Female	65-86 years	Widowed	Illiteracy	Housekeeper
432	61	Rural area	Female	50-64 years	Separated/Divorced	Illiteracy	Housekeeper
433	76	Rural area	Female	65-86 years	Single	Illiteracy	worker
434	37	Rural area	Female	21-49 years	Married/Cohabitation	Elementary	Housekeeper
435	68	Rural area	Female	65-86 years	Married/Cohabitation	Illiteracy	Housekeeper
436	56	Rural area	Female	50-64 years	Married/Cohabitation	Elementary	worker
437	58	Rural area	Men	50-64 years	Married/Cohabitation	Illiteracy	worker
438	66	Rural area	Men	65-86 years	Widowed	Illiteracy	Housekeeper
439	59	Rural area	Female	50-64 years	Married/Cohabitation	Elementary	Housekeeper
440	64	Rural area	Men	50-64 years	Married/Cohabitation	Illiteracy	worker
441	54	Rural area	Female	50-64 years	Married/Cohabitation	Illiteracy	worker
442	47	Rural area	Female	21-49 years	Single	Illiteracy	worker
443	53	Rural area	Female	50-64 years	Married/Cohabitation	Elementary	Housekeeper
444		Rural area	Female		Married/Cohabitation	Elementary	worker
445	66	Rural area	Female	65-86 years	Single	Elementary	Housekeeper
446	35	Rural area	Female	21-49 years	Married/Cohabitation	Elementary	Housekeeper
447	78	Rural area	Female	65-86 years	Married/Cohabitation	Elementary	Housekeeper
448	57	Rural area	Female	50-64 years	Single	Illiteracy	Housekeeper
449	58	Rural area	Female	50-64 years	Married/Cohabitation	Elementary	Housekeeper

450	42	Rural area	Female	21-49 years	Single	Elementary	Housekeeper
451	30	Rural area	Female	21-49 years	Married/Cohabitation	Elementary	Housekeeper
452	53	Rural area	Female	50-64 years	Widowed	Elementary	Housekeeper
453	37	Rural area	Female	21-49 years	Married/Cohabitation	Elementary	Housekeeper
454	71	Rural area	Men	65-86 years	Married/Cohabitation	Elementary	worker
455	55	Rural area	Female	50-64 years	Married/Cohabitation	Elementary	Housekeeper
456	38	Rural area	Female	21-49 years	Married/Cohabitation	Elementary	Housekeeper
457	37	Rural area	Female	21-49 years	Married/Cohabitation	Elementary	Housekeeper
458	30	Rural area	Men	21-49 years	Single	Elementary	worker
459	61	Rural area	Female	50-64 years	Married/Cohabitation	Elementary	Housekeeper
460	71	Rural area	Female	65-86 years	Married/Cohabitation	Elementary	worker
461	44	Rural area	Female	21-49 years	Married/Cohabitation	Elementary	Housekeeper
462	75	Rural area	Female	65-86 years	Married/Cohabitation	Illiteracy	Housekeeper
463	57	Rural area	Men	50-64 years	Married/Cohabitation	Elementary	
464	45	Rural area	Female	21-49 years	Married/Cohabitation	Elementary	Housekeeper
465	39	Rural area	Female	21-49 years	Separated/Divorced	Elementary	Housekeeper
466	48	Rural area	Female	21-49 years	Married/Cohabitation	Elementary	Housekeeper
467	72	Rural area	Female	65-86 years	Widowed	Elementary	Housekeeper
468	56	Rural area	Female	50-64 years	Married/Cohabitation	Elementary	Housekeeper
469	48	Rural area	Female	21-49 years	Married/Cohabitation	Elementary	Housekeeper
470	57	Rural area	Female	50-64 years	Married/Cohabitation	Elementary	Housekeeper
471	49	Rural area	Female	21-49 years	Married/Cohabitation	Elementary	Housekeeper
472	49	Rural area	Female	21-49 years	Widowed	Elementary	Housekeeper
473	51	Rural area	Female	50-64 years	Married/Cohabitation	Elementary	Housekeeper
474	50	Rural area	Female	50-64 years	Married/Cohabitation	Elementary	Housekeeper
475	66	Rural area	Female	65-86 years	Single	Elementary	worker
476	45	Rural area	Female	21-49 years	Married/Cohabitation	Elementary	Housekeeper
477	59	Rural area	Men	50-64 years	Married/Cohabitation	Elementary	worker
478	66	Rural area	Female	65-86 years	Widowed	Elementary	Housekeeper
479	62	Rural area	Female	50-64 years	Widowed	Illiteracy	worker

480	77	Rural area	Female	65-86 years	Widowed	Illiteracy	worker
481	44	Rural area	Female	21-49 years	Married/Cohabitation	Elementary	Housekeeper
482	54	Rural area	Female	50-64 years	Widowed	Illiteracy	Housekeeper
483	67	Rural area	Female	65-86 years	Married/Cohabitation	Elementary	Housekeeper
484	53	Rural area	Female	50-64 years	Widowed	Elementary	Housekeeper
485	63	Rural area	Female	50-64 years	Widowed	Elementary	Housekeeper
486	83	Rural area	Female	65-86 years	Widowed	Illiteracy	Housekeeper
487	63	Rural area	Female	50-64 years	Widowed	Illiteracy	Housekeeper
488	56	Rural area	Female	50-64 years	Separated/Divorced	Illiteracy	Housekeeper
489	63	Rural area	Female	50-64 years	Single	Illiteracy	Housekeeper
490	61	Rural area	Female	50-64 years	Widowed	Illiteracy	Housekeeper
491	68	Rural area	Men	65-86 years	Widowed	Illiteracy	worker
492	52	Rural area	Female	50-64 years	Married/Cohabitation	Elementary	Housekeeper
493	48	Rural area	Female	21-49 years	Widowed	Illiteracy	Housekeeper
494	66	Rural area	Female	65-86 years	Widowed	Illiteracy	worker
495	61	Rural area	Female	50-64 years	Widowed	Illiteracy	Housekeeper
496	64	Rural area	Female	50-64 years	Married/Cohabitation	Illiteracy	Housekeeper
497	42	Rural area	Female	21-49 years	Married/Cohabitation	Elementary	Housekeeper
498	51	Rural area	Female	50-64 years	Married/Cohabitation	Elementary	Housekeeper
499	60	Rural area	Female	50-64 years	Separated/Divorced	Elementary	Housekeeper
500	51	Rural area	Female	50-64 years	Married/Cohabitation	Elementary	Housekeeper
501	57	Rural area	Female	50-64 years	Married/Cohabitation	Elementary	Housekeeper
502	68	Rural area	Men	65-86 years	Married/Cohabitation	Elementary	worker
503	37	Rural area	Female	21-49 years	Married/Cohabitation	Elementary	Housekeeper
504	43	Rural area	Female	21-49 years	Married/Cohabitation	Elementary	Housekeeper
505	53	Rural area	Female	50-64 years	Separated/Divorced	Elementary	Housekeeper
506	56	Rural area	Female	50-64 years	Separated/Divorced	Elementary	Housekeeper
507	60	Rural area	Men	50-64 years	Married/Cohabitation	Elementary	worker
508	44	Rural area	Female	21-49 years	Single	Elementary	Housekeeper
509	55	Rural area	Female	50-64 years	Single	Elementary	Housekeeper

510	51	Rural area	Female	50-64 years	Married/Cohabitation	Elementary	Housekeeper
511	43	Rural area	Female	21-49 years	Married/Cohabitation	Elementary	Housekeeper
512	46	Rural area	Female	21-49 years	Married/Cohabitation	Elementary	Housekeeper
513	52	Rural area	Female	50-64 years	Widowed	Elementary	worker
514	49	Rural area	Female	21-49 years	Married/Cohabitation	Elementary	Housekeeper
515	55	Rural area	Men	50-64 years	Married/Cohabitation	Higher than elementary	worker
516	59	Rural area	Men	50-64 years	Married/Cohabitation	Elementary	worker
517	55	Rural area	Female	50-64 years	Married/Cohabitation	Elementary	Housekeeper
518	73	Rural area	Female	65-86 years	Married/Cohabitation	Elementary	worker
519	56	Rural area	Female	50-64 years	Married/Cohabitation	Elementary	Housekeeper
520	35	Rural area	Female	21-49 years	Single	Elementary	Housekeeper
521	53	Rural area	Female	50-64 years		Elementary	Housekeeper
522	47	Rural area	Female	21-49 years	Married/Cohabitation	Elementary	Housekeeper
523	44	Rural area	Female	21-49 years	Married/Cohabitation	Elementary	Housekeeper
524	65	Rural area	Female	65-86 years	Married/Cohabitation	Elementary	Housekeeper
525	60	Rural area	Female	50-64 years	Married/Cohabitation	Elementary	Housekeeper
526	68	Rural area	Female	65-86 years	Married/Cohabitation	Elementary	Housekeeper
527	47	Rural area	Female	21-49 years	Married/Cohabitation	Elementary	Housekeeper
528	46	Rural area	Female	21-49 years	Married/Cohabitation	Elementary	Housekeeper
529	55	Rural area	Female	50-64 years	Married/Cohabitation	Elementary	Housekeeper
530	42	Rural area	Female	21-49 years	Married/Cohabitation	Elementary	Housekeeper
531	61	Rural area	Female	50-64 years	Married/Cohabitation	Elementary	Housekeeper
532	47	Rural area	Female	21-49 years	Married/Cohabitation	Elementary	Housekeeper
533	53	Rural area	Men	50-64 years	Separated/Divorced	Elementary	worker
534	62	Rural area	Female	50-64 years	Widowed	Elementary	Housekeeper
535	37	Rural area	Female	21-49 years	Married/Cohabitation	Elementary	Housekeeper
536	55	Rural area	Female	50-64 years	Single	Elementary	Housekeeper
537	68	Rural area	Female	65-86 years	Single	Elementary	Housekeeper
538	53	Rural area	Female	50-64 years	Married/Cohabitation	Elementary	Housekeeper
539	56	Rural area	Female	50-64 years	Married/Cohabitation	Elementary	Housekeeper

540	50	Rural area	Female	50-64 years	Married/Cohabitation	Elementary	Housekeeper
541		Rural area	Female		Widowed	Elementary	worker
542	42	Rural area	Female	21-49 years	Married/Cohabitation	Elementary	Housekeeper
543	51	Rural area	Female	50-64 years	Married/Cohabitation	Elementary	Housekeeper
544	58	Rural area	Female	50-64 years	Married/Cohabitation	Illiteracy	Housekeeper
545	73	Rural area	Female	65-86 years	Widowed	Illiteracy	Housekeeper
546	68	Rural area	Female	65-86 years	Married/Cohabitation	Elementary	Housekeeper
547	48	Rural area	Female	21-49 years	Widowed	Elementary	Housekeeper
548	55	Rural area	Female	50-64 years	Married/Cohabitation	Elementary	worker
549	75	Rural area	Female	65-86 years	Married/Cohabitation	Elementary	Housekeeper
550	51	Rural area	Female	50-64 years	Married/Cohabitation	Elementary	Housekeeper
551	60	Rural area	Female	50-64 years	Married/Cohabitation	Elementary	Housekeeper
552	56	Rural area	Female	50-64 years	Married/Cohabitation	Elementary	Housekeeper
553	57	Rural area	Female	50-64 years	Married/Cohabitation	Elementary	Housekeeper

progrm	tdiab5	p31	comp_Neurc	comp_Amp	comp_Nefro	comp_Vista	comadbt	p89	Apoy_eco	Apoy_especi
1	3 years or fe\	0	0	0	0	0	0	no support		
	4-12 years	0	0	0	0	0	0	no support		
1	3 years or fe\	0	0	0	0	0	0	no support		
1	4-12 years	1		1				some support	1	0
1		0						no support		
1	3 years or fe\	0						some support	1	0
1	more than 12	1				1		some support	1	0
1	3 years or fe\	0	0	0	0	0	0	some support	1	0
1	4-12 years	0	0	0	0	0	0	some support	1	0
1	4-12 years	0	0	0	0	0	0	some support	1	0
1	3 years or fe\	0	0	0	0	0	0	some support	1	0
1	3 years or fe\	0	0	0	0	0	0	some support	0	0
1	3 years or fe\	0	0	0	0	0	0	some support	0	0
1	4-12 years	0	0	0	0	0	0	some support	1	0
1	3 years or fe\	0	0	0	0	0	0	some support	1	0
1	3 years or fe\	0	0	0	0	0	0	some support	1	0
1	4-12 years	0	0	0	0	0	0	some support	0	0
	3 years or fe\	0	0	0	0	0	0	some support	1	0
1	3 years or fe\	1				1		some support	1	1
1	3 years or fe\	0	0	0	0	0	0	some support	1	0
2	3 years or fe\	1				1		no support		
1	3 years or fe\	0	0	0	0	0	0	some support	0	0
1		1				1		some support	0	0
1	3 years or fe\	0	0	0	0	0	0	no support		
1	3 years or fe\	0	0	0	0	0	0	no support		
1	3 years or fe\	0	0	0	0	0	0	some support	1	0
1	more than 12	1				1		some support	1	0
1	more than 12	1				1	1	some support	1	1
1	4-12 years	0	0	0	0	0	0	no support		

1	more than 12	0						some support	0	0
1		0						no support		
1	3 years or fev	0						no support		
2	4-12 years	1				1	1	some support	0	0
1	more than 12	0	0	0	0	0	0	some support	1	0
1	3 years or fev	0	0	0	0	0	0	no support		
2	more than 12	1				1		no support		
1	more than 12	1				1		some support	0	0
2	3 years or fev	0	0	0	0	0	0	some support	0	0
	3 years or fev	0	0	0	0	0	0	some support	1	0
1	3 years or fev	0	0	0	0	0	0	some support	1	0
1		0						some support	1	0
1	4-12 years	0	0	0	0	0	0	some support	1	0
2	4-12 years	0	0	0	0	0	0	some support	0	0
1	4-12 years	1				1		no support		
2	3 years or fev	0	0	0	0	0	0	some support	1	0
1	more than 12	1				1		some support	1	1
2	more than 12	1				1		some support	1	0
2	3 years or fev	0	0	0	0	0	0	some support	1	0
	4-12 years	0	0	0	0	0	0	some support	1	0
1	4-12 years	0	0	0	0	0	0	some support	0	0
1	4-12 years	0	0	0	0	0	0	some support	0	0
2	3 years or fev	0	0	0	0	0	0	some support	1	0
1	3 years or fev	0	0	0	0	0	0	some support	1	0
1	3 years or fev	1				1		some support	0	0
2	4-12 years	1				1		some support	0	0
1		0	0	0	0	0	0	some support	1	0
1	3 years or fev	1				1		some support	1	1
1	4-12 years	0	0	0	0	0	0	some support	1	0
1	4-12 years	0						some support	1	1

1	3 years or fe	1				1		some support	1	0
1	4-12 years	0	0	0	0	0	0	some support	0	0
1	3 years or fe	0	0	0	0	0	0	some support	0	0
1	3 years or fe	0	0	0	0	0	0	some support	1	1
1	3 years or fe	0	0	0	0	0	0	some support	1	1
2	more than 12	1				1		some support	1	0
1	3 years or fe	0	0	0	0	0	0	no support		
1	4-12 years	0	0	0	0	0	0	no support		
1	4-12 years	0	0	0	0	0	0	no support		
1	3 years or fe	0	0	0	0	0	0	no support		
		0						no support		
1	3 years or fe	0	0	0	0	0	0	no support		
	more than 12	1				1				
		0								
2		0						some support	0	0
1	4-12 years	1	1			1		no support		
1		0						some support	0	0
2	more than 12	0						some support	1	0
1	3 years or fe	0	0	0	0	0	0	some support	1	0
2	3 years or fe	0	0	0	0	0	0	some support	1	1
1	more than 12	0								
1	3 years or fe	1				1		no support		
2	3 years or fe	0	0	0	0	0	0	no support		
1	3 years or fe	0						some support	0	0
1	3 years or fe	0	0	0	0	0	0	some support	1	0
1	4-12 years	0	0	0	0	0	0	some support	1	0
1	4-12 years	0	0	0	0	0	0	some support	0	0
1	3 years or fe	0	0	0	0	0	0	some support	1	1
1	3 years or fe	0	0	0	0	0	0	some support	0	0
1	3 years or fe	0	0	0	0	0	0	some support	1	0

1		0	0	0	0	0	0	some support	1	1
1	more than 12	0	0	0	0	0	0	some support	1	1
1	3 years or fev	0	0	0	0	0	0	some support	1	1
1	4-12 years	0	0	0	0	0	0	some support	1	1
1	4-12 years	0	0	0	0	0	0	some support	1	0
1	4-12 years	0	0	0	0	0	0	no support		
1	4-12 years	0	0	0	0	0	0	no support		
1	4-12 years	1				1		some support	0	0
1	3 years or fev	1				1		some support	1	1
1	4-12 years	1				1		no support		
1	more than 12	0	0	0	0	0	0	some support	0	0
1	more than 12	1				1		some support	1	0
1	more than 12	1		1				some support	1	0
1	4-12 years	1				1		some support	1	0
1	4-12 years	0						no support		
1	3 years or fev	1				1		some support	1	0
1	3 years or fev	0	0	0	0	0	0	some support	0	0
2	3 years or fev	0	0	0	0	0	0	no support		
1	more than 12	1				1		some support	0	0
2	4-12 years	1				1		some support	0	0
1	4-12 years	1				1		some support	0	0
1	4-12 years	1				1		no support		
1	4-12 years	0	0	0	0	0	0	some support	0	0
1	3 years or fev	0	0	0	0	0	0	some support	1	0
1	4-12 years	0	0	0	0	0	0	some support	1	0
1	4-12 years	1				1		some support	1	0
1	4-12 years	0						some support	0	0
1	4-12 years	1				1		some support	0	0
1	3 years or fev	0						some support	0	0
1	4-12 years	1	1					some support	1	1

1	3 years or fe	1		1				some support	0	0
1	4-12 years	0	0	0	0	0	0	no support		
1	more than 1	1			1			some support	1	0
1	4-12 years	0	0	0	0	0	0	no support		
1	4-12 years	0	0	0	0	0	0	no support		
1	more than 1	0	0	0	0	0	0	some support	1	1
1	4-12 years	0	0	0	0	0	0	no support		
1	3 years or fe	0						no support		
1	4-12 years	1				1		no support		
1	4-12 years	0	0	0	0	0	0	some support	0	0
1	3 years or fe	0	0	0	0	0	0	some support	1	0
1	more than 1	0	0	0	0	0	0	some support	0	0
1	4-12 years	0	0	0	0	0	0	some support	1	0
1	3 years or fe	1				1		some support	0	0
1	4-12 years	0						no support		
1	3 years or fe	0	0	0	0	0	0	some support	0	0
2	4-12 years	0	0	0	0	0	0	some support	0	0
1	3 years or fe	0	0	0	0	0	0	some support	0	0
2	3 years or fe	1				1		some support	1	0
2	4-12 years	1					1	some support	1	0
1	more than 1	0	0	0	0	0	0	some support	1	0
2	4-12 years	0	0	0	0	0	0	some support	1	0
1	4-12 years	0	0	0	0	0	0	some support	1	0
1	3 years or fe	1				1		some support	1	0
1	4-12 years	1	1					no support		
1	4-12 years	0	0	0	0	0	0	some support	1	0
	4-12 years	0	0	0	0	0	0	some support	0	0
1	4-12 years	0	0	0	0	0	0	no support		
2	3 years or fe	0	0	0	0	0	0	some support	1	0
1	4-12 years	0	0	0	0	0	0	no support		

2	4-12 years	0	0	0	0	0	0	no support		
1	4-12 years	1				1		some support	0	0
1	4-12 years	1				1		some support	1	0
1	3 years or fev	1				1		some support	1	0
1	4-12 years	0	0	0	0	0	0	no support		
1	3 years or fev	0	0	0	0	0	0	some support	1	0
2	4-12 years	0	0	0	0	0	0	some support	1	0
1	3 years or fev	0	0	0	0	0	0	no support		
2	4-12 years	0	0	0	0	0	0	no support		
2	4-12 years	1			1			some support	0	0
1	more than 12	0	0	0	0	0	0	some support	1	0
1	more than 12	1				1		some support	0	0
1	more than 12	0	0	0	0	0	0	some support	1	0
1	more than 12	0						some support	1	0
2	4-12 years	1				1		no support		
1	more than 12	1	1					some support	1	0
1	3 years or fev	0	0	0	0	0	0	some support	0	0
2	more than 12	0	0	0	0	0	0	some support	0	0
1	4-12 years	1			1			some support	1	0
1	3 years or fev	1	1			1		some support	0	0
2	4-12 years	0	0	0	0	0	0	some support	0	0
2	4-12 years	1					1	no support		
2	more than 12	1			1	1		no support		
2	more than 12	1			1			some support	0	0
2	4-12 years	1				1		some support	0	0
2	4-12 years	1				1		no support		
2	more than 12	0	0	0	0	0	0	some support	1	0
2	4-12 years	0	0	0	0	0	0	some support	1	0
1	more than 12	1			1	1		no support		
1	3 years or fev	0	0	0	0	0	0	no support		

2	4-12 years	0	0	0	0	0	0	no support		
2	3 years or fe\	0	0	0	0	0	0	some support	1	0
2	3 years or fe\	1	1					some support	1	0
2	4-12 years	0	0	0	0	0	0	some support	0	0
2	more than 1:	1				1		some support	0	0
2	4-12 years	1		1	1			some support	0	0
2	more than 1:	0	0	0	0	0	0	no support		
2	4-12 years	0	0	0	0	0	0	no support		
2	more than 1:	1				1		some support	1	0
2	more than 1:	1				1		some support	1	0
2	4-12 years	0	0	0	0	0	0	some support	0	0
2	4-12 years	1				1		no support		
2	4-12 years	0						no support		
2	4-12 years	0	0	0	0	0	0	no support		
2	more than 1:	0	0	0	0	0	0	no support		
2	3 years or fe\	0	0	0	0	0	0	some support	1	0
1	3 years or fe\	0	0	0	0	0	0	some support	0	0
1	4-12 years	1				1		no support		
2	4-12 years	0	0	0	0	0	0	some support	0	0
2	3 years or fe\	0	0	0	0	0	0	some support	0	0
2	more than 1:	1				1		no support		
2	more than 1:	1				1		no support		
2	more than 1:	0	0	0	0	0	0	no support		
1	4-12 years	0						some support	1	0
2	more than 1:	0	0	0	0	0	0	some support	0	0
1	more than 1:	0						some support	0	0
2	3 years or fe\	0	0	0	0	0	0	some support	1	0
2	more than 1:	1				1		some support	1	0
1	more than 1:	1					1	some support	1	0
1	more than 1:	0						some support	1	0

1	more than 12	0						no support		
2	more than 12	0						no support		
2	more than 12	1				1		some support	0	0
2	3 years or fev	1				1		some support	0	0
2	3 years or fev	0	0	0	0	0	0	some support	0	0
2	4-12 years	0						some support	0	0
2	4-12 years	0	0	0	0	0	0	some support	0	0
2	3 years or fev	0	0	0	0	0	0	some support	1	1
2	4-12 years	0	0	0	0	0	0	some support	0	0
2	4-12 years	0	0	0	0	0	0	some support	0	0
2	4-12 years	0	0	0	0	0	0	some support	0	0
1	4-12 years	0	0	0	0	0	0	some support	0	0
2	more than 12	0						some support	0	0
1	4-12 years	0	0	0	0	0	0	some support	1	1
1	more than 12	0						some support	0	0
2	more than 12	1				1		some support	0	0
2	more than 12	1					1	some support	0	0
2	4-12 years	1				1		some support	0	0
2	more than 12	0						some support	1	0
2	4-12 years	0						some support	1	0
2	4-12 years	1				1		some support	0	0
2	4-12 years	0						some support	0	0
	more than 12	0						some support	0	0
2	4-12 years	1				1		some support	0	0
2	4-12 years	0	0	0	0	0	0	no support		
2	4-12 years	0						some support	1	0
1	more than 12	0						some support	0	0
2	more than 12	1				1		some support	0	0
2	4-12 years	0	0	0	0	0	0	no support		
	4-12 years	0	0	0	0	0	0	some support	0	0

2	4-12 years	1				1		some support	1	1
2	4-12 years	0	0	0	0	0	0	no support		
2	3 years or fev	0	0	0	0	0	0	some support	1	0
2	4-12 years	1				1		some support	1	0
2	4-12 years	1				1		some support	0	0
2	3 years or fev	0	0	0	0	0	0	some support	0	0
2	4-12 years	1				1		some support	0	0
2	4-12 years	1				1		some support	0	0
2	more than 12	0	0	0	0	0	0	some support	0	0
2	more than 12	0	0	0	0	0	0	some support	1	0
2	more than 12	1				1	1	some support	1	0
2	more than 12	1				1		some support	0	0
2	more than 12	0	0	0	0	0	0	some support	1	0
1	3 years or fev	0						some support	0	0
2	more than 12	1				1		some support	1	0
1	more than 12	0	0	0	0	0	0	some support	0	0
2	4-12 years	0	0	0	0	0	0	some support	0	0
2	more than 12	0	0	0	0	0	0	no support		
1	3 years or fev	0	0	0	0	0	0	some support	1	1
1	3 years or fev	0	0	0	0	0	0	some support	0	0
1	4-12 years	0	0	0	0	0	0	some support	1	0
2	4-12 years	0	0	0	0	0	0	some support	0	0
2	more than 12	1				1		no support		
1	4-12 years	0	0	0	0	0	0	some support	1	0
1	4-12 years	0						some support	0	0
2	more than 12	0						some support	1	0
	3 years or fev	1	1					some support	0	0
1	more than 12	0						some support	0	0
1	4-12 years	1				1		some support	1	1
1	4-12 years	0	0	0	0	0	0	some support	1	0

2	4-12 years	0						some support	1	0
2	4-12 years	0						some support	1	1
2	4-12 years	0	0	0	0	0	0	some support	0	0
2	4-12 years	0	0	0	0	0	0	some support	1	0
1	3 years or fev	0	0	0	0	0	0	some support	0	0
2	4-12 years	1				1		some support	1	0
2	4-12 years	0	0	0	0	0	0	some support	0	0
2	4-12 years	0	0	0	0	0	0	some support	0	0
1	3 years or fev	0	0	0	0	0	0	some support	1	0
2	4-12 years	0						no support		
2	4-12 years	0	0	0	0	0	0	some support	1	0
1	more than 12	0	0	0	0	0	0	some support	0	0
1	4-12 years	0	0	0	0	0	0	some support	0	0
2	4-12 years	0	0	0	0	0	0	some support	1	0
1	3 years or fev	0	0	0	0	0	0	some support	0	0
2	4-12 years	1				1		some support	0	0
1	more than 12	0	0	0	0	0	0	some support	0	0
2	4-12 years	0	0	0	0	0	0	some support	0	0
2	more than 12	0	0	0	0	0	0	some support	1	0
1	4-12 years	0	0	0	0	0	0	some support	1	0
1	4-12 years	1				1		some support	1	0
2	4-12 years	0	0	0	0	0	0	some support	0	0
2	4-12 years	0	0	0	0	0	0	some support	0	0
1	more than 12	1	1			1		some support	0	0
1	4-12 years	0	0	0	0	0	0	some support	1	0
2	3 years or fev	0						some support	1	0
2	4-12 years	1	1			1		some support	1	1
2	more than 12	1				1		some support	1	0
2	3 years or fev	0	0	0	0	0	0	some support	0	0
1	3 years or fev	0	0	0	0	0	0	some support	1	0

2	more than 12	0	0	0	0	0	0	some support	1	0
2	4-12 years	1				1		some support	1	1
2	3 years or fev	0						some support	0	0
2	more than 12	1				1		some support	0	0
2	4-12 years	0	0	0	0	0	0	some support	0	0
2	4-12 years	0	0	0	0	0	0	some support	0	0
2	3 years or fev	0						some support	1	0
2	more than 12	0	0	0	0	0	0	some support	0	0
1	4-12 years	1				1		some support	0	0
2	4-12 years	1	1			1		some support	1	0
2	4-12 years	0	0	0	0	0	0	some support	1	0
2	4-12 years	0	0	0	0	0	0	some support	0	0
2	more than 12	1		1				some support	1	0
1	4-12 years	0	0	0	0	0	0	some support	1	1
1	4-12 years	0	0	0	0	0	0	some support	1	0
2	3 years or fev	0	0	0	0	0	0	some support	1	0
2	4-12 years	0						some support	1	1
2	more than 12	1				1		some support	0	0
1	3 years or fev	1	1					some support	0	0
2	more than 12	0	0	0	0	0	0	some support	0	0
2	3 years or fev	0	0	0	0	0	0	no support		
2	4-12 years	0	0	0	0	0	0	some support	1	1
1	more than 12	0	0	0	0	0	0	some support	0	0
2	more than 12	0	0	0	0	0	0	some support	0	0
1	more than 12	1				1		no support		
2	3 years or fev	0	0	0	0	0	0	no support		
2	4-12 years	1				1		no support		
2	more than 12	0	0	0	0	0	0	some support	1	1
2	more than 12	0	0	0	0	0	0	some support	0	0
2	4-12 years	1				1		some support	0	0

2	4-12 years	1				1		some support	0	0
2	3 years or fe	0	0	0	0	0	0	some support	1	0
1	3 years or fe	0						some support	0	0
2	more than 1	0	0	0	0	0	0	some support	1	0
2	3 years or fe	0	0	0	0	0	0	some support	1	0
1	4-12 years	0	0	0	0	0	0	some support	1	0
2	3 years or fe	0						some support	0	0
2	4-12 years	1				1		some support	0	0
2	4-12 years	0	0	0	0	0	0	some support	0	0
2	4-12 years	0	0	0	0	0	0	no support		
		0								
2	4-12 years	0	0	0	0	0	0	some support	1	1
2	4-12 years	0	0	0	0	0	0	some support	0	0
2	4-12 years	0	0	0	0	0	0	some support	1	0
1	4-12 years	0	0	0	0	0	0	no support		
2	4-12 years	1				1		some support	0	0
2	4-12 years	1		1		1		some support	0	0
2	3 years or fe	1				1		some support	1	0
2	more than 1	1				1		some support	1	0
2	more than 12 years							some support	1	1
2	3 years or fe	0	0	0	0	0	0	some support	1	0
2	more than 1	0	0	0	0	0	0	some support	0	0
2	4-12 years	0	0	0	0	0	0	some support	0	0
2	4-12 years	0	0	0	0	0	0	some support	0	0
2	4-12 years	0	0	0	0	0	0	some support	1	0
2	3 years or fe	0	0	0	0	0	0	some support	0	0
2	3 years or fe	0	0	0	0	0	0	some support	1	1
2	3 years or fe	0	0	0	0	0	0	no support		
2	4-12 years	0	0	0	0	0	0	no support		
2	3 years or fe	0	0	0	0	0	0	some support	1	0

1	more than 12	0	0	0	0	0	0	some support	1	0
2	more than 12	0						some support	1	0
2	more than 12	0	0	0	0	0	0	some support	1	0
1	4-12 years	0	0	0	0	0	0	no support		
1	4-12 years	0	0	0	0	0	0	some support	1	0
2	more than 12	0	0	0	0	0	0	some support	0	0
2	more than 12	0	0	0	0	0	0	no support		
1	3 years or few	0						some support	0	0
1	more than 12	1				1		some support	1	1
2	4-12 years	1				1		some support	0	0
2	more than 12	1				1		no support		
2	3 years or few	0						no support		
2	more than 12	0	0	0	0	0	0	some support	0	0
2	4-12 years	0	0	0	0	0	0	some support	1	1
2	4-12 years	0	0	0	0	0	0	some support	0	0
1	4-12 years	0	0	0	0	0	0	some support	0	0
2	more than 12	0	0	0	0	0	0	some support	1	0
2	4-12 years	0	0	0	0	0	0	some support	0	0
2	4-12 years	0						some support	1	0
2	4-12 years	0	0	0	0	0	0	some support	1	0
2	4-12 years	0	0	0	0	0	0	some support	0	0
2	more than 12	0	0	0	0	0	0	some support	0	0
2	3 years or few	0	0	0	0	0	0	some support	1	0
2	more than 12	0	0	0	0	0	0	some support	0	0
2	4-12 years	0	0	0	0	0	0	some support	1	0
2	more than 12	0	0	0	0	0	0	no support		
2	more than 12	0	0	0	0	0	0	some support	0	0
2	3 years or few	1		1				no support		
2	more than 12	1				1		some support	1	0
2	more than 12	0	0	0	0	0	0	some support	0	0

2	4-12 years	0						no support		
2	4-12 years	0	0	0	0	0	0	some support	0	0
2	4-12 years	1				1		some support	1	0
2	more than 12	0						some support	1	0
	4-12 years	0	0	0	0	0	0	some support	0	0
2	3 years or few	1				1		some support	1	1
	more than 12	1				1		some support	1	0
2	3 years or few	0	0	0	0	0	0	some support	0	0
2	4-12 years	0	0	0	0	0	0	some support	1	0
1	3 years or few	1				1		some support	0	0
2	more than 12	0	0	0	0	0	0	some support	0	0
1	4-12 years	0	0	0	0	0	0	no support		
2	4-12 years	0	0	0	0	0	0	some support	0	0
2	3 years or few	0	0	0	0	0	0	some support	1	0
2	more than 12	1				1		some support	1	0
2		0	0	0	0	0	0	no support		
2	4-12 years	0	0	0	0	0	0	some support	1	0
2	3 years or few	1	1					some support	0	0
2	4-12 years	1				1		some support	1	0
2	more than 12	0	0	0	0	0	0	some support	1	0
2	3 years or few	0						some support	0	0
2	4-12 years	0	0	0	0	0	0	some support	0	0
2	4-12 years	0	0	0	0	0	0	some support	0	0
2	4-12 years	1				1		some support	0	0
2	4-12 years	0	0	0	0	0	0	no support		
1	more than 12	0	0	0	0	0	0	some support	1	0
2	more than 12	0						no support		
2	4-12 years	1				1		some support	0	0
2	4-12 years	1				1		some support	0	0
2	4-12 years	1	1					some support	0	0

2	3 years or fe	0	0	0	0	0	0	some support	0	0
2	4-12 years	0	0	0	0	0	0	some support	1	0
2	3 years or fe	0	0	0	0	0	0	some support	0	0
2	4-12 years	0	0	0	0	0	0	some support	1	0
1	4-12 years	0	0	0	0	0	0	some support	1	0
2	more than 1	0	0	0	0	0	0	some support	1	0
1	4-12 years	0						some support	0	0
1	3 years or fe	0	0	0	0	0	0	some support	1	0
1	4-12 years	1	1					some support	0	0
1	more than 1	1					1	no support		
2	4-12 years	1				1		no support		
1	3 years or fe	0						some support	1	0
1	4-12 years	0	0	0	0	0	0	some support	0	0
2	3 years or fe	0	0	0	0	0	0	some support	1	0
1	4-12 years	1				1		some support	0	0
1	4-12 years	0	0	0	0	0	0	some support	1	1
1	3 years or fe	0	0	0	0	0	0	some support	1	0
2	4-12 years	1					1	some support	1	0
1	4-12 years	0	0	0	0	0	0	some support	0	0
2	more than 1	1				1		some support	1	0
1	4-12 years	0						some support	1	0
1	3 years or fe	0	0	0	0	0	0	some support	1	0
1	4-12 years	0	0	0	0	0	0	some support	0	0
2	3 years or fe	0	0	0	0	0	0	some support	1	0
2	4-12 years	0	0	0	0	0	0	some support	1	0
2	3 years or fe	0	0	0	0	0	0	some support	1	0
1	3 years or fe	0	0	0	0	0	0	some support	0	0
1	more than 1	0						some support	0	0
2	more than 1	1	1					some support	1	0
2	4-12 years	0	0	0	0	0	0	some support	1	0

1	3 years or fe	1				1		some support	1	1
1	4-12 years	0	0	0	0	0	0	some support	1	1
1	4-12 years	1				1		no support		
1	4-12 years	0	0	0	0	0	0	some support	1	0
1	more than 1	1				1		some support	1	0
1	3 years or fe	0	0	0	0	0	0	some support	0	0
1	3 years or fe	0	0	0	0	0	0	some support	1	1
1	3 years or fe	0	0	0	0	0	0	some support	0	0
2	3 years or fe	0	0	0	0	0	0	some support	1	0
1	4-12 years	0						some support	0	0
2	more than 1	1				1		some support	1	0
1	4-12 years	0	0	0	0	0	0	some support	0	0
1	3 years or fe	0	0	0	0	0	0	some support	1	0
		0								
1	3 years or fe	0	0	0	0	0	0	some support	0	0
1	4-12 years	1				1		some support	0	0
1	3 years or fe	0	0	0	0	0	0	some support	0	0
2	more than 1	1				1		no support		
2	4-12 years	0	0	0	0	0	0	some support	1	1
1	4-12 years	0						some support	0	0
1	4-12 years	0	0	0	0	0	0	some support	1	0
1	3 years or fe	1				1		some support	0	0
2	4-12 years	0						some support	1	0
1	3 years or fe	1				1		some support	1	0
1	more than 1	0						some support	0	0
2	more than 1	0	0	0	0	0	0	no support		
1	3 years or fe	0	0	0	0	0	0	some support	1	0
2	4-12 years	1				1		some support	1	0
2	more than 1	1				1		some support	1	0
2	more than 1	1				1		some support	1	0

1	more than 1	0	0	0	0	0	0	no support		
2	4-12 years	1				1		some support	0	0
2	3 years or fev	0	0	0	0	0	0	some support	0	0
2	3 years or fev	1				1		some support	1	1
2	4-12 years	0						no support		
2	more than 1	0	0	0	0	0	0	some support	0	0
2	more than 1	1				1		some support	1	0
2	more than 1	1				1		no support		
1	3 years or fev	0	0	0	0	0	0	some support	0	0
1	4-12 years	0	0	0	0	0	0	some support	1	0
1	3 years or fev	0	0	0	0	0	0	some support	0	0
1	more than 1	0	0	0	0	0	0	some support	1	1
2	4-12 years	1				1		some support	0	0
2	4-12 years	0	0	0	0	0	0	some support	1	0
2	4-12 years	1					1	some support	1	1
2	more than 1	1				1		some support	0	0
2	more than 1	0	0	0	0	0	0	some support	1	0
1	3 years or fev	0	0	0	0	0	0	some support	0	0
1		1				1		some support	0	0
2	4-12 years	1				1		some support	0	0
1	3 years or fev	1				1		some support	0	0
2	4-12 years	1				1		some support	1	1
2	4-12 years	0	0	0	0	0	0	some support	1	1
1	more than 1	1				1	1	no support		
1	more than 1	1				1		some support	1	0
2	4-12 years	0	0	0	0	0	0	some support	0	0
2	4-12 years	0	0	0	0	0	0	no support		
2	3 years or fev	0						some support	1	1
2	4-12 years	0						some support	1	0
2	4-12 years	1				1		some support	0	0

1	more than 1	0	0	0	0	0	0	some support	1	0
2	4-12 years	0	0	0	0	0	0	some support	0	0
2	3 years or fe	1	1	1				some support	1	1
2	more than 1	0						some support	0	0
1	more than 1	1				1		some support	0	0
1	4-12 years	0	0	0	0	0	0	some support	0	0
2	more than 1	1				1		some support	0	0
2	more than 1	1				1		no support		
2	3 years or fe	1				1	1	some support	1	1
2	4-12 years	1				1		some support	0	0
2	3 years or fe	0	0	0	0	0	0	some support	1	1
2	4-12 years	1				1		no support		
2	3 years or fe	0						some support	0	0
2	4-12 years	0	0	0	0	0	0	some support	1	0
2	more than 1	0	0	0	0	0	0	some support	1	0
2	more than 1	1				1		some support	0	0
1	more than 1	0	0	0	0	0	0	some support	0	0
1	3 years or fe	0						no support		
1	3 years or fe	0	0	0	0	0	0	some support	0	0
2	3 years or fe	1				1		some support	0	0
2	4-12 years	0						some support	0	0
2	4-12 years	1				1		some support	1	0
2	4-12 years	1				1		some support	0	0
2	4-12 years	1				1		some support	0	0
2	more than 1	1				1		some support	0	0
2	more than 1	1	1					some support	0	0
2	more than 1	1				1		some support	0	0
2		0						some support	0	0
1	4-12 years	1	1			1		some support	1	0
1		0								

1	more than 12	1				1		some support	1	1
2	more than 12	0						some support	1	0
1	4-12 years	0	0	0	0	0	0	some support	1	0
2	4-12 years	0						some support	0	0
2	more than 12	0						some support	0	0
1	4-12 years	0	0	0	0	0	0	some support	1	0
1	3 years or few	0						some support	0	0
1	4-12 years	1				1		some support	0	0
2	3 years or few	1	1			1		some support	0	0
1	4-12 years	0	0	0	0	0	0	some support	0	0
1	more than 12	0						no support		
2	more than 12	1				1		some support	0	0
2	4-12 years	0	0	0	0	0	0	some support	1	0
1	4-12 years	0	0	0	0	0	0	some support	0	0

Apoy_cuid	Apoy_afect	Apoy_otro	p57	p59	p85	p96	conocDM	scare	p43	trad2
			0	0	lives with relative	0	0	1	Yes	0
			0	0	lives with relative	1	0	0	Yes	0
			0	0	lives with relative	0	0	0	Yes	0
0	0	0	0	0	lives with relative	1	0	1	Yes	0
			0	0	lives with relative	0	0	0	Yes	0
0	0	0	0	0	lives with relative	0	0	0	Yes	0
0	0	0	0	0	lives with relative	1	0	0	Yes	0
0	0	0	0	0	lives with relative	1	0	0	Yes	0
0	0	0	0	0	lives with relative	0	0	0	Yes	0
0	0	0	0	0	lives alone	0	0	0	Yes	0
0	0	0	0	0	lives with relative	1	0	0	Yes	0
0	1	0	0	0	lives with relative	1	0	0	Yes	0
0	1	0	0	0	lives with relative	1	0	0	Yes	0
0	0	0	0	0	lives with relative	0	0	0	Yes	0
0	0	0	0	0	lives alone	0	0	0	Yes	0
0	0	0	0	0	lives with relative	1	0	0	Yes	0
0	1	0	0	0	lives with relative	1	0	0	regular	0
0	0	0	0	0	lives with relative	1	0	0	Yes	0
0	0	0	0	0	lives with relative	1	0	0	Yes	0
0	0	0	0	0	lives with relative	1	0	0	Yes	0
			0	0	lives with relative	1	0	0	Yes	0
1	0	0	0	0	lives with relative	1	0	0	Yes	0
0	1	0	0	0	lives with relative	0	0	0	Yes	0
			0	0	lives with relative	1	0	0	Yes	0
			1	1	lives with relative	1	0	0	Yes	0
0	0	0	0	0	lives with relative	1	0	0	Yes	0
0	0	0	0	0	lives with relative	1	0	1	Yes	0
0	0	0	0	0	lives with relative	0	0	0	Yes	0
			0	0	lives with relative	0	0	0	Yes	0

1	0	0	0	0	lives with relative	0	0	0	Yes	0
			0	0	lives with relative	1	0	0	regular	0
			0	0	lives with relative	1	0	0	Yes	0
1	0	0	0	0	lives with relative	0	0	0	Yes	0
0	0	0	0	0	lives with relative	1	0	0	Yes	0
			0	1	lives with relative	0	0	0	Yes	0
			0	1	lives with relative	0	0	0	Yes	0
0	0	1	0	0	lives with relative	1	0	0	Yes	0
0	1	0	0	0	lives alone	0	0	0	Yes	0
0	0	0	0	0	lives with relative	0	0	0	Yes	0
0	0	0	0	0	lives with relative	0	0	0	Yes	0
0	0	0	0	0	lives with relative	0	0	0	Yes	0
0	0	0	0	0	lives with relative	0	0	0	Yes	0
0	1	0	0	0	lives with relative	1	0	0	Yes	0
			0	0	lives alone	0	0	0	Yes	0
0	0	0	0	0	lives with relative	0	0	0	No	0
0	0	0	0	0	lives with relative	1	0	1	Yes	0
0	0	0	0	0	lives with relative	1	0	1	Yes	0
0	0	0	0	0	lives with relative	1	0	0	Yes	0
0	0	0	0	0	lives with relative	0	0	0	Yes	0
0	1	0	0	0	lives with relative	0	0	0	Yes	0
0	1	0	0	0	lives with relative	1	0	0	Yes	0
0	0	0	0	0	lives with relative	0	0	0	Yes	0
0	0	0	0	0	lives with relative	0	0	0	Yes	0
1	0	0	0	0	lives with relative	0	0	1	Yes	0
0	1	0	0	0	lives with relative	1	0	0	Yes	0
0	0	0	0	0	lives with relative	0	0	0	Yes	0
0	0	0	0	0	lives with relative	0	0	0	Yes	0
0	0	0	0	0	lives with relative	1	0	0	Yes	0
0	0	0	0	0	lives with relative	0	0	0	Yes	0

0	0	0	0	0	lives with relative	0	0	0	Yes	0
0	1	0	0	0	lives with relative	0	0	0	Yes	0
0	0	1	0	0	lives with relative	1	0	0	Yes	0
0	0	0	0	0	lives with relative	1	0	0	Yes	0
0	0	0	0	0	lives with relative	0	0	0	Yes	0
0	0	0	0	0	lives with relative	1	0	0	Yes	0
			0	0	lives with relative	1	0	0	Yes	0
			0	0	lives with relative	0	0	0	Yes	0
			0	0	lives with relative	0	0	0	Yes	0
			0	0	lives with relative	0	0	0	Yes	0
			0	0	lives with relative	1	0	0		0
			0	0	lives with relative	1	0	0	Yes	0
			1	0		0	0	0	Yes	0
			0	0	lives with relative	0	0	0		0
1	0	0	0	0	lives with relative	1	0	0		0
			0	0		0	0	0	Yes	0
0	0	1	0	0	lives with relative	1	0	0		0
0	0	0	0	0	lives with relative	0	0	0		0
0	0	0	0	0	lives with relative	1	0	0	Yes	0
0	0	0	0	1	lives with relative	1	0	1	Yes	0
			0	0	lives with relative	0	0	0	Yes	0
			0	0	lives with relative	0	0	0	Yes	0
			0	0	lives with relative	0	0	0	regular	0
1	0	0	0	0	lives with relative	1	0	0	regular	0
0	0	0	0	0	lives with relative	0	0	0	Yes	0
0	0	0	0	0	lives with relative	0	0	1	Yes	0
1	0	0	0	1	lives with relative	1	0	0	Yes	0
0	0	0	0	1	lives with relative	1	0	0	Yes	0
1	0	0	0	0	lives with relative	0	0	0	Yes	1
0	0	0	0	0	lives with relative	1	0	0	Yes	0

0	0	0	0	0	lives with relative	1	0	0	Yes	0
0	0	0	0	0	lives with relative	1	0	0	Yes	0
0	0	0	0	0	lives with relative	0	0	0	Yes	0
0	0	0	0	0	lives with relative	1	0	0	Yes	0
0	0	0	0	0	lives with relative	0	0	0	Yes	0
			0	0	lives with relative	0	0	0	Yes	0
			0	0	lives with relative	0	0	0	Yes	0
0	1	0	0	0	lives with relative	0	0	0	regular	0
0	0	0	0	0	lives alone	1	0	0	Yes	0
			0	0	lives with relative	1	0	0	Yes	0
1	0	0	0	0	lives with relative	0	0	0	Yes	0
0	0	0	0	0	lives with relative	0	0	0	Yes	0
0	0	0	0	0	lives with relative	0	0	0	Yes	0
0	0	0	0	0	lives with relative	1	0	0	Yes	0
			0	0	lives alone	1	0	0	Yes	0
0	0	0	0	0	lives with relative	0	0	0	Yes	0
1	0	0	0	0	lives with relative	0	0	0	Yes	0
			0	0	lives with relative	1	0	0	Yes	0
1	0	0	0	0	lives with relative	1	0	0	Yes	0
1	0	1	0	0	lives with relative	0	0	0	Yes	0
0	1	0	0	0	lives with relative	0	0	0	Yes	0
			0	0	lives with relative	1	0	0	Yes	0
1	0	0	0	0	lives with relative	1	0	0	Yes	0
0	0	0	0	0	lives with relative	1	0	0	regular	0
0	0	0	1	1	lives with relative	1	0	0	Yes	0
0	0	0	0	0	lives with relative	1	0	0	Yes	0
1	0	0	0	0	lives with relative	0	0	0	Yes	0
0	1	0	0	0	lives with relative	0	0	0	Yes	0
1	0	0	0	0	lives with relative	0	0	0	Yes	0
0	0	0	0	0	lives with relative	0	0	0	Yes	0

1	0	0	0	0	lives with relative	1	0	0	regular	0
			0	0	lives with relative	1	0	0	Yes	0
0	0	0	0	0	lives with relative	1	0	0	Yes	0
			0	0	lives with relative	1	0	0	Yes	0
			0	0	lives with relative	1	0	0	Yes	0
0	0	0	0	1	lives with relative	1	0	1	Yes	0
			0	0	lives with relative	0	0	0	Yes	0
			0	0	lives with relative	1	0	0	Yes	0
			0	0	lives alone	0	0	0	Yes	0
0	1	0	0	0	lives with relative	1	0	0	Yes	0
0	0	0	0	0	lives with relative	1	0	0	Yes	0
1	0	0	0	0	lives with relative	0	0	0	Yes	0
0	0	0	0	0	lives with relative	0	0	0	Yes	0
0	1	0	0	0	lives with relative	0	0	0		0
			0	0	lives alone	0	0	0	Yes	0
0	1	0	0	0	lives with relative	0	1	1	Yes	0
0	1	0	0	0	lives with relative	0	0	0		0
1	0	0	0	0	lives with relative	1	0	0	Yes	0
0	0	0	0	0	lives alone	1	0	0	Yes	0
0	0	0	0	0	lives with relative	0	0	0	Yes	0
0	0	0	0	0	lives with relative	1	0	0	Yes	0
0	0	0	0	1	lives with relative	1	1	0	Yes	0
0	0	0	0	0	lives with relative	0	0	0	Yes	0
0	0	0	0	0	lives with relative	1	0	0	Yes	0
			0	0	lives with relative	0	0	0	Yes	0
0	0	0	0	0	lives with relative	1	0	0	Yes	0
1	0	0	0	0	lives with relative	0	0	0	Yes	0
			0	0	lives with relative	0	0	0	Yes	0
0	0	0	0	0	lives with relative	1	0	1	Yes	0
			0	0	lives with relative	1	0	0	Yes	0

			0	0	lives with relative	0	0	0	Yes	0
1	0	0	0	0	lives with relative	0	0	0	Yes	0
0	0	0	0	0	lives with relative	1	0	1	Yes	0
0	0	0	0	0	lives with relative	0	0	0	Yes	0
			0	0	lives with relative	1	0	0	Yes	0
0	0	0	0	0	lives with relative	0	0	0	Yes	1
0	0	0	0	0	lives with relative	0	0	0	Yes	0
			0	0	lives with relative	0	0	0	Yes	0
			0	0	lives with relative	1	0	0	Yes	0
1	0	0	1	0	lives with relative	1	0	0	Yes	0
0	0	0	0	0	lives with relative	1	0	1	regular	0
0	0	1	0	0	lives with relative	0	0	0	Yes	0
0	0	0	0	0	lives with relative	0	0	0	Yes	0
0	0	0	0	0	lives with relative	1	0	0	Yes	0
			0	0	lives with relative	1	0	0	No	0
0	0	0	0	0	lives alone	0	0	0	Yes	0
1	0	0	0	0	lives with relative	0	0	0	Yes	0
1	0	0	0	0	lives with relative	1	0	0	Yes	0
0	0	0	0	0	lives with relative	1	0	0	Yes	0
1	0	0	0	0	lives with relative	1	0	0	Yes	0
1	0	0	0	1	lives with relative	1	0	0	regular	0
			0	0	lives with relative	1	0	0	Yes	0
			0	0	lives with relative	1	0	0	Yes	0
1	0	0	0	0	lives with relative	1	0	0	Yes	0
1	0	0	0	0	lives with relative	0	0	0	Yes	0
			0	1	lives with relative	0	0	0	Yes	0
0	0	0	0	1	lives with relative	0	0	0	Yes	0
0	0	0	0	0	lives with relative	1	0	0	Yes	0
			0	0	lives with relative	1	0	0	Yes	0
			0	0	lives with relative	0	0	0	Yes	0

			0	1	lives with relative	0	0	0	Yes	0
0	0	0	0	0	lives with relative	1	0	0	Yes	0
0	0	0	0	0	lives with relative	1	0	0	Yes	1
1	0	0	0	0	lives with relative	0	0	0	Yes	0
0	1	0	0	0	lives with relative	1	0	0	Yes	0
0	1	0	0	0	lives with relative	1	0	0	Yes	0
			0	0	lives with relative	1	0	0	Yes	0
			0	0	lives with relative	0	0	0	Yes	0
0	0	0	0	0	lives with relative	1	0	0	Yes	0
0	0	0	0	0	lives with relative	1	0	0	Yes	0
0	1	0	0	0	lives with relative	1	0	0	Yes	0
			0	0	lives alone	1	0	0	Yes	0
			0	0	lives with relative	1	0	0	Yes	0
			0	1	lives with relative	0	0	0	Yes	0
			0	0	lives with relative	1	0	0	Yes	0
0	0	0	0	0	lives with relative	0	0	0	Yes	0
1	0	0	0	0	lives with relative	1	0	0	Yes	0
			0	0	lives with relative	1	0	0	Yes	0
1	0	0	0	0	lives with relative	1	0	0	Yes	0
1	0	0	0	0	lives with relative	1	0	0	Yes	0
			0	0	lives with relative	1	0	0	Yes	0
			1	0	lives with relative	1	0	0	Yes	1
			0	0	lives with relative	1	0	0	Yes	0
0	0	0	0	0	lives with relative	1	0	0	Yes	0
0	1	0	0	0	lives with relative	1	0	0	Yes	0
0	1	0	0	0	lives with relative	0	0	0	Yes	0
0	0	0	0	0	lives with relative	1	0	0	Yes	0
0	0	0	0	0	lives alone	0	0	0	Yes	0
0	0	0	0	0	lives with relative	1	0	0	Yes	0
0	0	0	0	0	lives with relative	1	0	0	Yes	0

			0	0	lives alone	1	0	0	regular	1
			0	0	lives with relative	0	0	0	regular	0
1	0	0	0	0	lives with relative	0	0	0	Yes	0
1	0	0	0	0	lives with relative	1	0	0	Yes	0
1	0	0	1	1	lives with relative	0	0	0	Yes	0
0	0	1	0	0	lives with relative	0	0	0	No	0
0	0	1	0	0	lives with relative	1	0	0	Yes	0
0	0	0	0	0	lives with relative	1	0	0	Yes	0
1	0	0	0	1	lives with relative	1	0	0	regular	0
1	0	0	0	1	lives with relative	1	0	0	Yes	0
0	0	1	0	0	lives with relative	1	0	0	Yes	0
1	0	0	0	0	lives with relative	0	0	0	Yes	0
0	0	1	0	0	lives with relative	1	0	0	Yes	0
0	0	0	0	0	lives with relative	0	0	1	Yes	0
1	0	0	0	0	lives with relative	1	0	0	Yes	0
0	1	0	0	0	lives with relative	1	0	0	Yes	0
1	0	0	0	1	lives with relative	1	0	1	Yes	0
1	0	0	0	0	lives with relative	1	0	0	Yes	1
0	0	0	1	1	lives with relative	1	0	0	Yes	0
0	0	0	0	0	lives with relative	1	0	0	Yes	0
1	0	0	0	1	lives with relative	1	0	0		0
0	0	1	1	0	lives alone	1	0	0	Yes	0
1	0	0	0	0	lives with relative	0	0	0	Yes	0
0	1	0	0	0	lives with relative	1	0	0	Yes	0
			0	0	lives with relative	0	0	0	Yes	0
0	0	0	0	0	lives with relative	1	0	0	Yes	0
1	0	0	0	0	lives alone	1	0	0	No	1
1	0	0	0	0	lives with relative	1	0	0	regular	0
			0	0	lives with relative	0	0	1	Yes	0
0	1	0	0	1	lives alone	1	0	1	Yes	0

0	0	0	0	0	lives with relative	1	0	0	Yes	0
			0	0	lives with relative	1	0	0	Yes	0
0	0	0	0	0	lives with relative	0	0	0	Yes	0
0	0	0	0	0	lives with relative	0	0	0	regular	0
0	1	0	0	0	lives alone	1	0	0	No	0
0	1	0	0	0	lives with relative	0	0	0	Yes	0
1	0	0	0	1	lives with relative	1	0	0	Yes	0
0	1	0	0	1	lives with relative	1	0	0	regular	0
1	0	0	0	0	lives with relative	0	0	1	Yes	0
0	0	0	0	0	lives with relative	1	0	0	Yes	0
0	0	0	0	0	lives with relative	0	0	0	Yes	0
0	1	0	0	0	lives alone	1	0	0	Yes	0
0	0	0	0	0	lives with relative	1	0	0	Yes	0
0	1	0	0	0	lives with relative	0	0	0	regular	0
0	0	0	0	0	lives with relative	1	0	0	Yes	0
0	0	1	0	0	lives with relative	1	0	0	Yes	0
1	0	0	1	0	lives with relative	1	1	0	Yes	0
			0	0	lives with relative	0	0	0	Yes	0
0	0	0	0	0	lives with relative	1	0	0	Yes	0
0	1	0	0	0	lives with relative	1	0	0	Yes	0
0	0	0	0	0	lives with relative	1	0	0	Yes	0
1	0	0	0	1	lives with relative	1	0	0	Yes	0
			0	0	lives alone	1	0	0	Yes	0
0	0	0	0	0	lives alone	1	0	0	Yes	1
1	0	0	0	1	lives with relative	1	0	0	Yes	0
0	0	0	1	0	lives with relative	1	0	0	Yes	0
0	1	0	0	0	lives with relative	1	0	0	Yes	0
0	1	0	0	0	lives with relative	1	0	0	Yes	0
0	0	0	0	0	lives with relative	0	0	0	Yes	0
0	0	0	0	0	lives with relative	1	0	0	Yes	1

0	0	0	0	0	lives with relative	1	0	0	Yes	0
0	0	0	0	0	lives with relative	0	0	0	regular	0
0	1	0	0	0	lives with relative	1	0	0	regular	0
0	0	0	0	0	lives with relative	0	0	0	Yes	0
1	0	0	0	0	lives with relative	1	0	0	Yes	0
0	0	0	0	0	lives with relative	1	0	0	Yes	0
0	1	0	1	0	lives with relative	1	0	0	Yes	0
0	1	0	0	0	lives with relative	1	0	0	Yes	0
0	0	0	0	0	lives with relative	1	0	0	Yes	0
			0	0	lives with relative	0	0	0	Yes	0
0	0	0	0	0	lives with relative	1	0	0	No	0
0	1	0	0	0	lives with relative	1	0	0	Yes	0
0	1	0	0	0	lives with relative	1	0	0	Yes	0
0	0	0	1	0	lives with relative	1	0	0	Yes	0
0	1	0	0	0	lives with relative	1	0	0	Yes	0
0	1	0	0	0	lives with relative	1	0	0	regular	0
0	1	0	0	0	lives with relative	0	0	0	Yes	0
0	1	0	0	0	lives with relative	1	0	0	Yes	0
0	0	0	1	1	lives with relative	1	0	0	Yes	0
0	0	0	0	0	lives with relative	1	0	0	regular	0
0	0	0	0	0	lives with relative	1	0	0	Yes	0
0	1	0	0	0	lives with relative	1	0	0	Yes	0
0	1	0	1	0	lives with relative	1	0	0	Yes	0
0	1	0	0	0	lives with relative	1	0	0	Yes	1
0	0	0	0	0	lives with relative	0	0	0	Yes	0
0	0	0	0	0	lives with relative	1	0	0	Yes	0
0	0	0	0	0	lives with relative	1	0	0	Yes	0
0	0	0	0	0	lives with relative	1	0	0	Yes	0
0	1	0	0	0	lives with relative	1	0	0	Yes	0
0	0	0	0	0	lives with relative	0	0	0	Yes	0

0	0	0	1	0	lives alone	1	0	0	Yes	0
0	0	0	0	0	lives with relative	1	0	0	Yes	0
1	0	0	0	0	lives with relative	1	0	0	Yes	0
1	0	0	0	0	lives with relative	1	0	0	Yes	0
1	0	0	0	0	lives with relative	0	0	0	regular	0
1	0	0	0	0	lives with relative	0	0	0	Yes	1
0	0	0	0	0	lives alone	1	0	0	Yes	0
0	1	0	0	0	lives with relative	1	0	0	Yes	0
0	1	0	0	0	lives with relative	0	0	0	Yes	0
0	0	0	0	0	lives with relative	1	0	0	Yes	0
0	0	0	1	1	lives with relative	1	0	0	Yes	0
0	1	0	0	0	lives with relative	1	0	0	Yes	0
0	0	0	0	1	lives with relative	0	0	0	Yes	0
0	0	0	0	1	lives alone	1	0	0	Yes	0
0	0	0	1	1	lives with relative	1	0	0	Yes	0
0	0	0	0	0	lives with relative	1	0	0	Yes	0
0	0	0	0	1	lives with relative	1	0	0	Yes	0
1	0	0	1	0	lives with relative	1	0	0	regular	0
1	0	0	0	0	lives with relative	1	0	0	Yes	0
0	1	0	0	0	lives with relative	1	0	0	Yes	0
			0	0	lives with relative	1	0	0	Yes	0
0	0	0	0	0	lives with relative	1	0	0	Yes	0
0	1	0	0	0	lives with relative	1	0	0	Yes	0
0	0	1	0	0	lives with relative	1	0	0	Yes	0
			0	0	lives with relative	1	0	0	Yes	0
			0	0	lives with relative	1	0	0	Yes	0
			0	0	lives with relative	1	0	0	Yes	0
0	0	0	0	0	lives with relative	1	0	0	Yes	0
1	0	0	0	0	lives with relative	1	0	0	Yes	0
1	0	0	0	0	lives with relative	1	0	0	Yes	0

1	0	0	0	0	lives with relative	1	0	0	Yes	1
0	0	0	0	1	lives alone	1	0	0	Yes	0
1	0	0	0	0	lives with relative	1	0	0	regular	0
0	0	0	0	0	lives with relative	1	0	0	Yes	0
0	0	0	0	0	lives with relative	1	0	0	Yes	0
0	0	0	1	0	lives with relative	1	1	0	Yes	0
0	1	0	0	0	lives with relative	1	0	0	Yes	0
0	0	1	1	0	lives with relative	1	0	0	Yes	0
0	1	0	0	0	lives with relative	1	0	0	Yes	0
			0	0	lives with relative	0	0	0	Yes	0
			0	0		0	0	0		0
0	0	0	1	0	lives with relative	1	0	0	Yes	0
1	0	0	0	0	lives with relative	1	0	0	Yes	0
0	0	0	0	0	lives with relative	1	0	0	Yes	0
			0	0	lives alone	1	0	0	Yes	0
1	0	0	0	0	lives with relative	1	0	0	Yes	0
0	1	0	0	0	lives alone	1	0	0	Yes	0
0	0	0	0	0	lives with relative	1	0	0	Yes	0
0	0	0	0	0	lives with relative	0	0	0	Yes	0
0	0	0	0	0	lives with relative	1	0	0	Yes	0
0	0	0	1	0	lives with relative	1	0	0	Yes	0
0	1	0	1	0	lives with relative	1	0	0	Yes	0
0	1	0	0	0	lives with relative	1	0	0	Yes	0
0	1	0	0	0	lives with relative	1	1	0	Yes	0
0	0	0	0	0	lives with relative	0	0	0	Yes	0
0	1	0	0	0	lives with relative	1	0	0	Yes	0
0	0	0	0	0	lives with relative	1	0	0	Yes	0
			0	0	lives with relative	1	0	0	Yes	0
			0	0	lives with relative	1	0	0	Yes	0
0	0	0	0	0	lives with relative	0	0	0	Yes	0

0	0	0	0	0	lives with relative	1	0	0	Yes	0
0	0	0	0	0	lives with relative	1	0	0	Yes	0
0	0	0	0	0	lives with relative	1	0	0	Yes	0
			0	0	lives with relative	1	0	0	regular	0
0	0	0	0	0	lives with relative	1	0	0	Yes	0
1	0	0	1	0	lives with relative	1	0	0	Yes	0
			0	0	lives with relative	1	1	0	Yes	0
0	1	0	0	0	lives with relative	1	0	0	Yes	0
0	0	0	0	0	lives alone	1	0	0	Yes	0
1	0	0	0	0	lives with relative	1	0	0	Yes	0
			0	0	lives with relative	0	0	0	Yes	0
			0	0	lives alone	1	0	0	Yes	0
1	0	0	0	0	lives with relative	1	0	0	Yes	0
0	0	0	0	0	lives with relative	1	0	0	Yes	0
1	0	0	0	0	lives with relative	1	0	0	Yes	0
0	1	0	0	0	lives with relative	1	0	0	regular	0
0	0	0	0	0	lives with relative	0	0	0	Yes	0
0	1	0	0	0	lives with relative	1	0	0	Yes	0
0	0	0	0	0	lives with relative	0	0	0	Yes	0
0	0	0	0	0	lives with relative	1	0	0	Yes	0
0	1	0	1	1	lives with relative	1	0	0	Yes	0
1	0	0	0	0	lives with relative	1	0	0	Yes	0
0	0	0	0	0	lives with relative	1	0	0	No	0
1	0	0	0	0	lives with relative	1	0	0	Yes	0
0	0	0	0	0	lives with relative	0	0	0	Yes	0
			0	0	lives with relative	1	0	0	Yes	0
1	0	0	0	0	lives with relative	1	0	0	Yes	0
			0	0	lives with relative	0	0	0	Yes	0
0	0	0	0	0	lives alone	1	1	0	Yes	0
0	1	0	0	0	lives with relative	1	0	0	Yes	0

			0	0	lives with relative	1	0	0	Yes	0
0	1	0	0	0	lives with relative	1	0	0	Yes	0
0	0	0	0	0	lives with relative	0	0	0	regular	0
0	0	0	0	0	lives with relative	1	0	0	Yes	0
1	0	0	0	0	lives with relative	1	0	0	Yes	0
0	0	0	0	0	lives with relative	1	0	0	Yes	0
0	0	0	0	0	lives alone	1	0	0	Yes	0
0	1	0	0	0	lives with relative	1	0	0	Yes	0
0	0	0	0	0	lives alone	1	0	0	regular	0
1	0	0	0	0	lives with relative	1	0	0	Yes	0
0	1	0	0	0	lives with relative	1	0	0	Yes	0
			0	1	lives alone	1	0	0	Yes	0
0	1	0	0	0	lives with relative	1	0	0	regular	0
0	0	0	0	0	lives with relative	1	0	0	Yes	0
0	0	0	0	0	lives with relative	1	0	0	Yes	0
			0	0	lives alone	1	0	0	regular	0
0	0	0	0	0	lives with relative	1	0	0	No	0
1	0	0	0	0	lives with relative	1	0	0	Yes	0
0	0	0	0	0	lives with relative	1	0	0	Yes	0
0	0	0	0	0	lives with relative	1	0	0	Yes	0
1	0	0	0	0	lives with relative	1	0	0	Yes	0
1	0	0	0	0	lives with relative	1	0	0	Yes	0
0	1	0	0	0	lives with relative	1	0	0	Yes	0
1	0	0	0	0	lives with relative	1	0	0	Yes	0
			0	0	lives with relative	0	0	0	Yes	0
0	0	0	0	0	lives with relative	1	0	0	Yes	0
			0	0	lives with relative	1	0	0	No	0
1	0	0	0	0	lives with relative	1	0	0	Yes	0
1	0	0	0	0	lives with relative	1	0	0	Yes	0
0	1	0	0	1	lives alone	1	0	0	Yes	0

1	0	0	0	0	lives with relative	1	0	0	Yes	0
0	0	0	0	0	lives with relative	1	0	0	Yes	0
1	0	0	0	0	lives with relative	1	0	0	Yes	0
0	0	0	0	0	lives with relative	1	0	0	Yes	0
0	0	0	0	0	lives with relative	1	0	1	Yes	0
0	0	0	0	0	lives with relative	1	0	0	Yes	0
1	0	0	0	0	lives with relative	1	0	0	Yes	0
0	0	0	0	0	lives with relative	1	0	0	Yes	0
0	1	0	0	0	lives with relative	1	0	1	Yes	0
			0	0	lives alone	1	0	0	Yes	0
			0	0	lives alone	0	0	0	Yes	0
0	0	0	0	0	lives with relative	0	0	0	Yes	0
1	0	0	0	0	lives with relative	0	0	0	regular	0
0	0	0	1	0	lives with relative	0	0	0	Yes	1
1	0	0	0	0	lives with relative	1	0	0	Yes	0
0	0	0	0	0	lives with relative	1	0	0	Yes	0
0	0	0	0	0	lives with relative	0	0	0	Yes	0
0	0	0	0	0	lives with relative	1	0	0	Yes	0
1	0	0	0	0	lives with relative	0	0	0	Yes	0
0	0	0	0	0	lives with relative	1	0	0	Yes	0
0	0	0	0	0	lives with relative	1	0	0	Yes	0
0	0	0	0	0	lives with relative	1	0	0	Yes	0
1	0	0	0	0	lives with relative	1	0	0	Yes	0
0	0	0	0	0	lives with relative	1	0	0	regular	0
0	0	0	0	0	lives with relative	1	0	0	Yes	0
0	0	0	0	0	lives with relative	1	0	0	Yes	0
0	1	0	0	0	lives with relative	1	0	0	Yes	0
0	1	0	0	0	lives with relative	0	0	1	Yes	0
0	0	0	0	0	lives with relative	1	0	1	Yes	0
0	0	0	0	0	lives with relative	1	0	0	Yes	0

0	0	0	0	0	lives with relative	1	0	0	Yes	0
0	0	0	0	0	lives with relative	1	0	0	Yes	0
			0	0	lives with relative	1	0	0	Yes	0
0	0	0	0	0	lives with relative	1	0	0	Yes	0
0	0	0	0	0	lives with relative	1	0	0	Yes	0
1	0	0	1	0	lives with relative	0	0	0	Yes	0
0	0	0	0	0	lives with relative	1	0	1	Yes	0
1	0	0	0	0	lives with relative	1	0	0	Yes	0
0	0	0	0	0	lives with relative	1	0	0	Yes	0
1	0	0	0	0	lives with relative	1	0	0	Yes	0
0	1	0	0	0	lives with relative	1	0	1	Yes	0
1	0	0	0	0	lives with relative	1	0	0	Yes	0
0	0	0	0	0	lives with relative	1	0	0	Yes	0
			0	0		0	0	0		0
0	1	0	0	0	lives with relative	1	0	0	Yes	0
1	0	0	0	0	lives with relative	1	0	0	Yes	0
0	1	0	0	0	lives with relative	1	0	0	Yes	0
			0	0	lives with relative	0	0	0	Yes	0
0	0	0	0	0	lives with relative	1	0	0	Yes	0
0	1	0	0	0	lives with relative	1	0	1	Yes	0
0	0	0	0	0	lives with relative	1	1	1	Yes	0
0	1	0	0	0	lives with relative	0	0	1	Yes	0
0	0	0	0	0	lives with relative	0	0	0	Yes	0
0	0	0	0	0	lives with relative	1	0	0	Yes	0
0	1	0	0	0	lives with relative	0	0	0	Yes	0
			0	0	lives with relative	1	0	0	Yes	0
0	0	0	0	0	lives with relative	1	0	0	Yes	0
0	0	0	0	0	lives with relative	1	0	0	Yes	0
0	0	0	0	0	lives alone	1	0	0	Yes	0
0	0	0	0	0	lives alone	1	0	0	Yes	0

			0	1	lives alone	0	0	0	Yes	0
0	1	0	0	0	lives with relative	1	0	0	Yes	0
1	0	0	0	0	lives alone	0	0	0	Yes	0
0	0	0	0	0	lives with relative	1	0	1	Yes	0
			0	0	lives with relative	1	0	0	Yes	0
1	0	0	0	0	lives with relative	1	0	1	Yes	0
0	0	0	0	0	lives alone	1	0	0	Yes	0
			0	0	lives with relative	1	0	1	Yes	0
1	0	0	0	1	lives with relative	1	0	0	regular	0
0	0	0	0	0	lives alone	1	0	0	Yes	0
1	0	0	0	0	lives with relative	1	0	0	Yes	0
0	0	0	0	0	lives with relative	0	0	0	Yes	0
0	1	0	0	0	lives with relative	1	0	0	regular	0
0	0	0	0	0	lives with relative	0	0	0	Yes	0
0	0	0	0	0	lives with relative	0	0	0	Yes	0
1	0	0	0	0	lives with relative	1	0	0	Yes	0
0	0	0	0	1	lives with relative	0	0	0	Yes	0
0	1	0	0	0	lives with relative	1	0	1	Yes	0
0	1	0	0	0	lives with relative	1	0	1	Yes	0
0	1	0	0	1	lives with relative	1	0	0	regular	0
0	1	0	0	0	lives with relative	1	0	0	Yes	0
0	0	0	0	0	lives with relative	1	0	0	Yes	0
0	0	0	0	1	lives with relative	1	0	1	Yes	0
			0	0	lives with relative	0	0	1	Yes	0
0	0	0	0	1	lives with relative	1	0	0	Yes	0
1	0	0	0	0	lives alone	1	1	0	Yes	0
			0	0	lives alone	1	0	0	Yes	0
0	0	0	1	1	lives with relative	0	0	0	Yes	0
0	0	0	0	0	lives with relative	1	0	0	Yes	0
0	1	0	0	0	lives with relative	0	0	0	regular	0

0	0	0	0	0	lives with relative	1	0	1	Yes	0
1	0	0	0	1	lives with relative	1	0	0	Yes	0
0	0	0	0	0	lives with relative	1	0	0	Yes	0
1	0	0	0	0	lives with relative	1	0	0	Yes	0
1	0	0	0	0	lives with relative	1	0	0	Yes	0
1	0	0	0	0	lives with relative	1	0	0	Yes	0
0	1	0	1	1	lives with relative	1	0	0	regular	0
			0	0	lives with relative	1	0	0	Yes	0
0	0	0	0	0	lives with relative	1	0	0	Yes	0
0	1	0	0	0	lives with relative	1	0	1	Yes	0
0	0	0	0	0	lives with relative	1	1	1	Yes	0
			0	0	lives with relative	1	0	0	Yes	0
0	1	0	0	0	lives with relative	1	0	1	regular	0
0	0	0	0	0	lives with relative	1	0	0	regular	0
0	0	0	0	0	lives with relative	1	0	0	Yes	0
0	1	0	0	0	lives with relative	0	0	0	Yes	0
0	1	0	0	0	lives with relative	1	0	0	Yes	0
			0	0	lives with relative	0	0	0	Yes	0
1	0	0	0	1	lives with relative	1	0	0	Yes	0
1	0	0	0	0	lives with relative	0	0	1	Yes	0
1	0	0	0	0	lives with relative	1	0	1	Yes	0
0	0	0	0	0	lives with relative	1	1	0	Yes	0
1	0	0	0	0	lives with relative	1	0	1	Yes	0
0	0	1	0	0	lives with relative	1	0	0	Yes	0
0	1	0	0	0	lives with relative	1	0	1	Yes	0
1	0	0	0	0	lives with relative	1	0	0	Yes	0
1	0	0	0	0	lives with relative	1	0	0	Yes	0
1	0	0	0	0	lives with relative	0	0	0		0
0	0	0	0	0	lives with relative	0	0	0	Yes	0
			0	0	lives with relative	0	0	0	Yes	0

0	0	0	0	0	lives with relative	1	0	0	Yes	0
0	0	0	0	0	lives with relative	1	0	0	Yes	0
0	0	0	0	1	lives with relative	1	0	0	Yes	0
0	1	0	0	0	lives with relative	1	0	0	Yes	0
1	0	0	0	0	lives with relative	1	0	0	Yes	0
0	0	0	0	0	lives with relative	0	0	0	Yes	0
1	0	0	0	0	lives with relative	1	0	0	Yes	0
1	0	0	0	0	lives with relative	1	0	0	Yes	0
1	0	0	0	0	lives with relative	1	0	0	Yes	0
1	0	0	0	0	lives with relative	1	0	0	regular	0
			0	0	lives alone	1	0	0	Yes	0
1	0	0	0	0	lives with relative	1	0	0	Yes	0
0	0	0	0	0	lives with relative	0	0	0	Yes	0
0	1	0	0	0	lives with relative	1	0	1	Yes	0
